# Preparation and Evaluation of Niosomal *Clerodendrum serratum* (Linn.) Moon Extract Formulations: Comparative In Silico and In Vitro Studies of Drying Methods for the Treatment of Hemorrhoids

**DOI:** 10.1155/sci5/3662572

**Published:** 2026-04-29

**Authors:** Anunya Suksanga, Komgrit Eawsakul, Kingkan Bunluepuech

**Affiliations:** ^1^ Research Excellence Center for Innovation and Health Products (RECIHP), Walailak University, Nakhon Si Thammarat, 80160, Thailand, wu.ac.th; ^2^ Department of Applied Thai Traditional Medicine, School of Medicine, Walailak University, Nakhon Si Thammarat, 80160, Thailand, wu.ac.th; ^3^ School of Allied Health Sciences, Walailak University, Nakhon Si Thammarat, 80160, Thailand, wu.ac.th

## Abstract

**Background:**

The pain and suffering of patients with hemorrhoids are strongly impacted by the inadequacy of available treatment options, with more severe cases facing suboptimal outcomes. Owing to its known properties, the traditional Thai medicine *Clerodendrum serratum* (Linn.) Moon (CSM) served as the cornerstone for this investigational study.

**Methods:**

To validate and scrutinize the extent of the therapeutic effects of CSM, we compared two drying techniques: a dehydration chamber and a hot air oven. Analysis of computational biology, otherwise known as “network pharmacology,” was employed to hypothesize and validate key pharmacological targets and investigate the toxicity and anti‐inflammatory properties of CSM in L929 and RAW 264.7 cells. Furthermore, this study presents the development of a niosome‐based enhanced therapeutic delivery formulation for CSM.

**Results and Discussion:**

Inhibiting the production of nitric oxide (NO) demonstrated that CSM dehydration performed in the dehydration chamber resulted in an IC_50_ of 8.50 ± 0.39 μg/mL, while the hot air oven method was cytotoxic (15.29 ± 3.17 μg/mL). The anti‐inflammatory response has been linked to the following key genes: NF‐κB1, PTGS2, ALOX5, EGFR, and NFE2L2. For maximum yield and encapsulation efficiency, the optimal Span 60:Tween 80: Cholesterol ratio was 11.0:19.0:13.5.

**Conclusions:**

The data indicate that CSM extract, especially when dehydrated in a dehydration chamber and then encapsulated in niosomes, has the potential to serve as a safe and effective therapeutic for hemorrhoids. The findings also emphasize the role of the drying method in retaining the bioactivity of plant extracts and the need for niosomes as a delivery system to improve bioavailability.

## 1. Introduction

Hemorrhoids are an anorectal disorder, causing various symptoms, including abnormal swelling of vascular tissues in the anal canal, and leading to bleeding, chronic inflammation, pain, bacterial infection, itching, and prolapse. These symptoms can significantly affect patients’ quality of life. Moreover, the incidence of hemorrhoids is 4.0%–55.0% of the global population [1]. Hemorrhoids can be classified into 4 stages, ranging from mild to severe symptoms [2, 3]. Furthermore, mild hemorrhoids can progress to severe hemorrhoids because of prolonged strain to defecate, chronic constipation or diarrhea, obesity, pregnancy, and a sedentary lifestyle, as well as aging [4].

The current treatments for hemorrhoids depend on the grade of symptoms. In the mild stage, treatment comprises dietary and lifestyle modification or medication, whereas in the severe stage, surgical interventions are needed [5, 6]. However, these treatments always have side effects [7, 8]. Therefore, alternative treatments need to be developed. Furthermore, several studies have reported that hemorrhoids are associated with inflammation, characterized by increased levels of cytokines and enzymes such as TNF‐α, nitric oxide (NO), inducible NO synthase (iNOS), MMPs, IL‐6, and IL‐17 [9, 10].

Dysregulation of T‐helper (Th) cell–mediated immune responses, particularly Th2 dominance, showed a role in chronic inflammatory conditions by promoting cytokine release, tissue remodeling, and persistent inflammation, as reported in former mechanistic studies on allergic asthma [11]. Consistent with this immunopathological mechanism, *Mesua ferrea* L. has been reported to exert therapeutic effects in allergic asthma by modulating cytokine production in asthmatic rat models, further supporting the potential of medicinal plants in regulating immune‐mediated inflammatory responses [12].

In addition, the pathology of rectal tissues reveals a large number of inflammatory cells. Consequently, a decrease in inflammatory cells is a main indicator of successful treatment.

*Clerodendrum serratum Linn. Moon* (CSM), a plant species belonging to the Lamiaceae family, has gained significant attention in traditional medicine systems. Various parts of CSM have been used to treat a wide range of conditions, including respiratory disorders, inflammatory diseases, skin ailments, and hemorrhoids, in regions such as Africa, Southeast Asia, Malaysia, and Thailand [13]; [14]. In traditional Thai medicine, CSM has a long history of being used to treat hemorrhoids, for example, inserting burned leaves into the rectum or administering leaf powder to reduce hemorrhoid size, while the stem and root, combined with lime water, can be applied externally to shrink hemorrhoids [15]. Solapure et al. [16] reported the anti‐inflammatory effects of 8.64 mL/kg *C. serratum* and *S. xanthocarpum* plants in Wistar albino rats. Moreover, recent studies of *C. serratum* further support the anti‐inflammatory and immunomodulatory potential, demonstrating that ethanolic extract of *C. serratum* significantly attenuated inflammatory mediator production in an ovalbumin‐induced asthma rat model [17]. Moreover, evidence from related bioactive compounds supports the effect of immune modulation in asthma. For instance, solasodine, a steroidal glycoalkaloid, has been reported to suppress exaggerated Th2‐mediated immune responses in ovalbumin‐induced asthma [18]. However, the efficacy and underlying mechanisms of CSM, specifically for treating hemorrhoids, remain unclear. In addition, CSM extract has a short therapeutic window and high cellular toxicity.

Niosomes are nanoparticles that have amphiphile properties [19, 20]. In the present study, niosomes were employed as carriers with the objective of improving the delivery of a CSM‐derived compound through encapsulation. As Singh et al. [21] reported, two‐layer niosomes can better deliver drugs because they can grab the extracted compound at the lipophilic site and let it dissolve in water at the hydrophilic site. The stability of niosomes can be enhanced by incorporating Span, Tween, ethoxylated alcohols, sucrose esters, and cholesterol [22, 23]. The stability of the niosome can prolong compound activity by slowing the release of the active compound [24, 25]. Network pharmacology and in silico target prediction approaches are increasingly applied in drug discovery to identify potential molecular targets and elucidate mechanisms of action, particularly for multicomponent herbal medicines [26]. Target prediction and reverse screening tools facilitate early‐stage hypothesis generation by linking bioactive compounds with disease‐related targets prior to experimental validation [27].

Therefore, the aim of this study is to validate and compare the effectiveness of CSM prepared using two drying methods: a hot air oven and a dehydration chamber. In addition, we employed network pharmacology, a computational approach that involves mapping complex biological networks to predict critical target genes and assess the toxicity and anti‐inflammatory effects of CSM on L929 (mouse fibroblast) and RAW 264.7 (mouse macrophage) cells.

Finally, we aimed to increase the therapeutic time of CSM extraction by determining the optimum conditions for the niosome.

## 2. Materials and Methods

### 2.1. Materials

SwissTargetPrediction, SuperPred, SEA Search, OMIM, and GeneCards are free online web servers for searching for compound targets and relevant genes for hemorrhoids. Cytoscape was used to analyze the string network.

L929 and RAW 264.7 macrophage lines were purchased from ATCC (Manassas, VA, USA). The murine fibroblast line L929 (NCTC clone 929) was obtained from subcutaneous connective tissue (ATCC CCL‐1). Blood monocytes were the source of the murine macrophage line RAW 264.7 (ATCC TIB‐71). *Mus musculus* was the source of both cell lines. The ATCC does not specify the sex of origin. These cell lines were chosen because they are commonly used in studies of inflammation and cytotoxicity (ISO 10993‐5).

3‐(4,5‐Dimethylthiazol‐2‐yl)‐2,5‐diphenyltetrazolium bromide (MTT), lipopolysaccharide (LPS), and Griess reagent were purchased from Invitrogen (Waltham, USA). The culture media, including fetal bovine serum (FBS), Dulbecco’s modified Eagle’s medium (DMEM), Dulbecco’s phosphate‐buffered saline (DPBS), trypan blue stain, and 0.25% trypsin EDTA, were purchased from Gibco (CA, USA).

Materials to prepare the nanoparticle niosomes, namely, Span 60, Tween 80, and cholesterol, were purchased from Sigma‐Aldrich (St. Louis, USA). Centrifugal filters were obtained from Fisher Scientific (Vantaa, Finland). All the solvents were purchased from RCI Labscan Ltd. (Bangkok, Thailand).

### 2.2. Plant Materials

CSM was identified by the Department of Thai Traditional and Alternative Medicine. Leaves were collected in 2023 from Ban Ko, Phrom Khiri District, Nakhon Si Thammarat Province, Thailand, and a voucher sample (SKP 072,021,901) was deposited. The collection procedures adhered to the relevant guidelines and regulations established by the Plant Varieties Protection, Department of Agriculture, Ministry of Agriculture and Cooperatives, Thailand. All experiments were performed in accordance with the biosafety guidelines for scientific research at Walailak University, Nakhon Si Thammarat, Thailand (ref. no. WU‐IBC‐67‐072).

### 2.3. Drying and Preparation of Samples

In this study, two leaf extracts were prepared using different drying techniques, and their characteristics were evaluated to determine how the drying method influences the antihemorrhoidal activity of the plant. One group was dried in a hot air oven at 70°C for 16 h, while the other was placed in a dehydration chamber maintained at 67% humidity and 25°C for 72 h. Both groups were dried until their water activity value (*a*_*w*_) fell below 0.6. The dried leaves were then ground and soaked in 95% ethanol for 14 days. Afterward, the ethanol was removed using a rotary evaporator, and the extract was frozen at −20°C before use.

### 2.4. Investigation of Chemical Compounds by Liquid Chromatography (LC)–Quadrupole Time‐of‐Flight (QTOF) MS/MS

The chemical composition of CSM was analyzed using LC‐QTOF MS/MS. This technique is very high‐performing because of the combination of LC, electrospray ionization (ESI), QTOF, and mass spectrometry (MS). HPLC separation was performed on an Agilent 1260 Infinity Series HPLC system equipped with a Luna C‐18 column. Linear gradient elution of water and acetonitrile, both containing 0.1% formic acid, was used. The mobile phase composition was gradually changed from 5% to 95% water over 30 min, followed by a 10 min re‐equilibration phase. A 10 μL sample was injected at a flow rate of 0.5 mL/min. MS data were collected using an Agilent 6540 QTOF mass spectrometer in both positive and negative ESI modes, with a mass‒charge ratio range of 100–1000 Da. Agilent Mass Hunter software was used for data analysis.

### 2.5. Screening of Components and Target Prediction

LC‐ESI‐QTOF MS/MS analysis revealed various compounds within CSM. Compounds with a library score exceeding 90 points were chosen as potential active compounds for further investigation [28]. Following the selection of the active compound, all three target prediction web servers, namely, SwissTargetPrediction (https://www.swisstargetprediction.ch/index.php; accessed on 26 April 2024) [29], SuperPred (https://prediction.charite.de/; accessed on 14 June 2024), and the SEA Search server (https://sea.bkslab.org/; accessed on 14 June 2024), were used to analyze every compound identified in CSM. The aim of this search was to predict potential gene targets for each compound within CSM. The analysis focused specifically on genes from the human species (*Homo sapiens*) to identify potential targets for the active compound.

### 2.6. Prediction of Hemorrhoid Targets

To identify genes potentially involved in hemorrhoid development, two online gene databases were searched: GeneCards (https://www.genecards.org/; accessed on 30 April 2024) and OMIM (https://www.omim.org/; accessed on 30 April 2024). These databases included only human genes in *Homo sapiens* and provided detailed information about their function. These terms were used to search for inflammation, oxidants, and infection that are related to the effects of hemorrhoids. The results from GeneCards and OMIM were subsequently combined and filtered to remove duplicates and analyzed to identify potential gene targets that are relevant to hemorrhoid treatment.

### 2.7. Construction of a Target Gene−Based Network

In this study, Venn diagrams (https://bioinformatics.psb.ugent.be/webtools/Venn/; accessed on 25 October 2024) were used to identify genes from both the CSM analysis and the hemorrhoid gene list. These overlapping genes demonstrate potential target genes for the active compound in CSM, suggesting that they might be influenced by the compound and potentially play a role in hemorrhoid development. Next, Cytoscape software (Version 3.8.2) was used to create a network visualization of the interacting genes. The STRING database was used to retrieve known and predicted interactions between the identified genes. This visual network helps us understand the relationships between these genes. Furthermore, the CytoHubba plugin within Cytoscape was used to identify the top 10 most interconnected compound genes within the network (accessed on 25 October 2024). These genes, with the highest degree values, are considered core genes and are likely to play a central role in the network of interacting genes potentially associated with hemorrhoids.

### 2.8. Cytotoxicity Test on the L929 Cell Line

L929 murine fibroblasts were cultured in flasks containing DMEM supplemented with 10% FBS. The culture conditions were maintained at 37°C with 5% CO_2_. Cytotoxicity was assessed according to ISO 10993‐5:2009(en) (Biological evaluation of medical devices ‐ Part 5: Tests for in vitro cytotoxicity [30]). L929 cells were seeded into a 96‐well plate at a density of 1 × 10^6^ cells per 100 μL of DMEM. The study groups included a negative control group (culture medium only) and experimental groups treated with various concentrations of CSM extract (7.81–1000 μg/mL), with each concentration tested in triplicate. After 24 h of incubation, the cells were washed with culture medium, and the CSM extract was added to each well.

After a 2‐h treatment period, cell viability was assessed using the MTT assay. A blue–purple color change in the supernatant following the addition of DMSO indicated the dissolution of formazan, a positive marker of cell viability. The absorbance of each well was measured at 570 nm using a microplate reader (Metertech, Taipei, Taiwan). This measurement was used to calculate cell viability, reported as the mean ± standard deviation (SD). Samples with cell viability percentages less than 70% were classified as cytotoxic.

### 2.9. Anti‐Inflammatory Effects on the RAW 264.7 Cell Line

RAW 264.7 macrophages derived from mice were used in this study. The study groups included a positive control group (treated with LPS), a negative control group (untreated), and experimental groups treated with various concentrations of CSM extract (1.95–500 μg/mL). Each concentration was tested in triplicate. Cells were seeded at a density of 1 × 10^6^ cells per well. After 24 h of incubation, the culture medium was replaced with phenol red−free DMEM, and LPS (1 μg/mL) was added to the positive control and experimental groups. The cells were allowed to produce NO for an additional 24 h. The CSM extract was then added to the LPS‐activated cells. A 100 μL aliquot of culture supernatant was mixed with an equal volume of Griess reagent and incubated for 10 min [31]. A pink color change in 202, the supernatant, indicated NO production. The absorbance was measured at 570 nm and 203 nm, and the results are reported as mean ± SDs. The percentage of NO inhibition 204 was calculated as

(1) % NO  inhibition=ODcontrol−ODsampleODcontrol×100.

### 2.10. Cell Viability Assays on RAW 264.7 Cell Line

The RAW 264.7 macrophage line exhibited sustained viability throughout the duration of the investigation. All the study groups, including the control groups, exhibited similar results in the anti‐inflammatory test. Cells were seeded at a density of 1 × 10^6^ cells per well and incubated for 24 h. The culture medium was replaced with phenol red−free DMEM, and LPS (1 μg/mL) was added to induce NO production. After a 24‐h incubation period, the extracted CSM was added to the activated cells. The culture supernatant was removed, and MTT reagent was added to each well. Cell viability was assessed by measuring formazan formation, which resulted in a blue–purple color change in the supernatant. The absorbance was measured at 570 nm using a microplate reader (Metertech, Taipei, Taiwan).

### 2.11. Preparation of CSM‐Loaded Niosomes

Span 60, Tween 80, and cholesterol were used as surfactant components in the thin‐film hydration process to create niosomes. With the use of the Box–Behnken design (BBD) created in Minitab software (Version 21.0), the three compounds were dissolved in 10 mL of chloroform and combined at different concentrations ranging from 5 to 20 mg/10 mL. After 10 mg of CSM extract was added to the mixture, the solvent was removed to create a thin lipid layer. To obtain evenly distributed niosomes, the film was rehydrated with 10 mL of distilled water and sonicated for three minutes at 80% amplitude. Until additional analysis, the formulations were kept at 4°C. Three independent factors were included in the BBD: cholesterol (X_3_), Tween 80 (X_2_), and Span 60 (X_1_). Each of these variables had three coded levels (−1, 0, +1), which corresponded to 5.0, 12.5, and 20.0 mg per 10 mL, respectively. To assess pure error, 15 experimental runs were conducted, including 3 center‐point replicates (Figure S13). The following fixed requirements were met for every run: 10 mg of CSM extract, 10 mL of chloroform, 3 min of 80% amplitude probe sonication, and 10 min of centrifugation at 1500 rpm. The yield percentage and encapsulation efficiency (% EE) were the measured answers. Analysis of variance (ANOVA) was used to assess the impacts of linear, quadratic, and interaction factors as well as the significance of the model.

### 2.12. Particle Size and Charge of CSM‐Loaded Niosomes

The rehydrated niosome solution was diluted with deionized water prior to measurement. Size and zeta potential were measured using a Malvern Zetasizer (Malvern Instruments, UK) with the dynamic light scattering (DLS) technique. Three measurements per sample were taken at 25°C. The measurements were performed at a wavelength of 663 nm, a scattering angle of 173°, a medium viscosity of 0.8872 cP, and a refractive index of 1.33 [22].

### 2.13. Determination of Yield, EE, and Drug Loading Content

The niosome solution was centrifuged at 1800 rpm for 30 min at 4°C to separate the free drug from the encapsulated drug. Both the niosome solution and the free drug solution were collected, and the dried solutions were freeze‐dried. After drying for approximately 24 h, the niosome powder and free drug powder were weighed to determine the yield (Equation (2)). To determine theEE and drug loading content, the niosome powder was dissolved in 95% ethanol. The drug concentration was then measured by determining the absorbance at 412 nm. TheEE and drug loading content were calculated using Equations 3 and 4, respectively [32–34].

(2) % Yield=weight of the nanoparticletheoretical total amount of nanoparticle×100,

(3) % DLC=amount of encapsulated CSM in niosomeweight of the nanoparticle×100,

(4) % EE=amount of encapsulated CSM in niosomeinitial amount of CSM×100.

### 2.14. Statistical Examination

The data are reported as mean ± SDs. Statistical analyses were conducted using one‐way ANOVA with Duncan’s post hoc test to compare differences between the experimental groups using SPSS software (Version 18.0). Values were considered statistically significant when the p value was less than 0.05.

## 3. Results and Discussion

### 3.1. Production of the Extracted CSM

#### 3.1.1. LC‐MS Profile of the Extracted CSM

The dehydration chamber (shade) drying process resulted in a CSM yield approximately 2.55% greater than that of the hot air oven method (Table 1).

**TABLE 1:** Yields for each step of *Clerodendrum serratum* (Linn.) Moon (CSM) extracted production.

Process	Drying process			
	Hot air oven		Dehydration chamber (shade)	
	Weight (g)	Yield (%)	Weight (g)	Yield (%)
1. Removing petiole	666.91	100.00	437.36	100.00
2. Drying	186.37	27.95	162.23	37.09
3. Milling	185.34	27.79	159.20	36.40
4. Extracting	12.16	1.82	19.11	4.37

LC‐MS analysis was performed on ethanol extracts from CSM samples dried using both hot air oven and dehydration chamber (shade) methods. LC‐MS/MS analysis was carried out using ESI^+^ and ESI^−^ modes with MS/MS acquisition. Compounds listed in Tables S1–S4 were selected based on a matching score of > 90 and supported by accurate mass and MS/MS fragmentation data.

Compounds for further investigation were selected on the basis of two key factors: the data identification score (> 90%) and the compound content (> 1.0%). The hot air oven‐dried CSM sample exhibited six peaks at retention times of 2.00, 3.77, 7.94, 9.41, 11.20, and 30.62 min (Table 2). Fragmentation of these peaks yielded spectra with candidate masses (m/z) of 192.06, 354.10, 464.10, 448.10, 446.09, and 294.22. Interpretation using the Spectrum Database for Organic Compounds (SDBS) application revealed quinic acid, chlorogenic acid (CGA), quercetin 3‐galactoside, kaempferol‐7‐o‐glucoside, baicalin, and 9(S)‐HOTrE.

**TABLE 2:** LC‐MS profile of ethanol extracts of *Clerodendrum serratum* (Linn.) Moon (CSM) drying in a hot air oven.

No.	m/z	RT (min)	Name of the compound	Molecular formula	Molecular weight	% Content
1	191.057	2.00	Quinic acid	C_7_H_12_O_6_	192.06	2.68
2	353.088	3.77	Chlorogenic acid	C_16_H_18_O_9_	354.10	1.27
3	463.088	7.94	Quercetin 3‐galactoside	C_21_H_20_O_12_	464.10	1.03
4	449.108	9.41	Kaempferol‐7‐o‐glucoside	C_21_H_20_O_11_	448.10	1.07
5	445.078	11.20	Baicalin	C_21_H_18_O_11_	446.09	1.35
6	293.213	30.62	9(S)‐HOTrE	C_18_H_30_O_3_	294.22	1.31

The major constituents in hot‐air oven‐dried CSM were predominantly flavonoids and their derivatives (quercetin 3‐galactoside, baicalin, and kaempferol‐7‐o‐glucoside), phenolic acids (quinic acid and CGA), and a long‐chain fatty acid (9(S)‐HOTrE).

Different drying methods resulted in varying compound profiles. LC‒MS analysis of ethanol extracts from CSM dried in a dehydration chamber revealed five peaks at retention times of 9.21, 9.41, 17.06, 17.48, and 32.23 min (Table 3). Fragmentation of these peaks yielded spectra with candidate masses (m/z) of 162.03, 448.10, 784.46, 270.05, and 292.21. Interpretation using the SDBS application revealed 4‐hydroxycoumarin, astragalin, Astragaloside IV, genistein, and 9‐OxoOTrE. All compounds obtained from both the hot‐air oven and dehydration chamber (shade) drying methods were selected on the basis of an identification score greater than 90%, as shown in Tables S1–S2 and the LC‐MS chromatograms in Figures S1–S4.

**TABLE 3:** LC‐MS profile of ethanol extracts of *Clerodendrum serratum* (Linn.) Moon (CSM) drying in the dehydration chamber.

No.	m/z	RT (min)	Name of the compound	Molecular formula	Molecular weight	% Content
1	163.039	9.21	4‐Hydroxycoumarin	C_9_H_6_O_3_	162.03	2.20
2	449.109	9.41	Astragalin	C_21_H_20_O_11_	448.10	1.05
3	783.455	17.06	Astragaloside IV	C_41_H_68_O_14_	784.46	1.06
4	271.060	17.48	Genistein	C_15_H_10_O_5_	270.05	1.64
5	291.198	32.23	9‐OxoOTrE	C_18_H_28_O_3_	292.21	2.47

Although both drying methods yielded a peak at approximately 9.41 min (m/z 448.10), the MS/MS fragmentation data indicated the presence of positional isomers of kaempferol glycosides. In the hot‐air oven‐dried CSM, the compound was tentatively annotated as kaempferol‐7‐O‐glucoside, whereas in the dehydration chamber–dried CSM, the corresponding peak was annotated as astragalin (kaempferol‐3‐O‐glucoside). These compounds share the same molecular formula and precursor mass but differ in the position of glycosylation (C‐7 vs. C‐3), which may result in very similar chromatographic behavior and nearly identical retention times. Accordingly, the observed similarity in RT is most reasonably attributed to co‐elution of positional isomers rather than chemical transformation. This clarification has been incorporated into the Results section, and the supporting MS/MS spectra are provided in Supporting Figures S5–S6 and S14–S15.

#### 3.1.2. Predicting Potential Target Genes of CSM in the Treatment of Hemorrhoids

The hemorrhoid‐related genes from GeneCards and OMIM were compared with the target genes associated with each active component of CSM dried using a hot air oven or dehydration chamber. The overlap of target genes between CSM dried using a hot air oven and that dehydrated in a chamber was found to be between 28 and 33 similar target genes. These common target genes are considered prospective targets for CSM in the treatment of hemorrhoids, as shown in Figures 1(a) and 1(b).

FIGURE 1The potential target genes of CSM were dried in a hot air oven (a) and in a dehydration chamber (b) with hemorrhoids.

**Figure:**
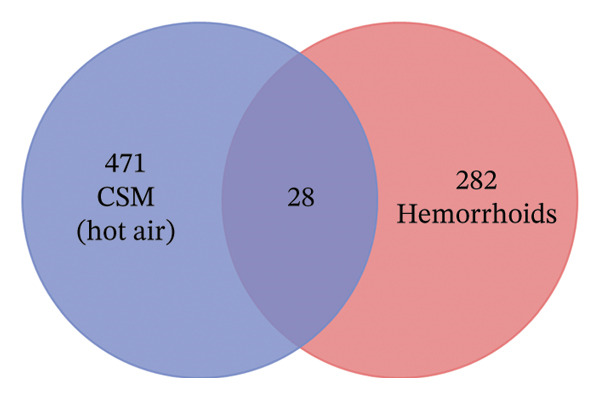
(a)

**Figure:**
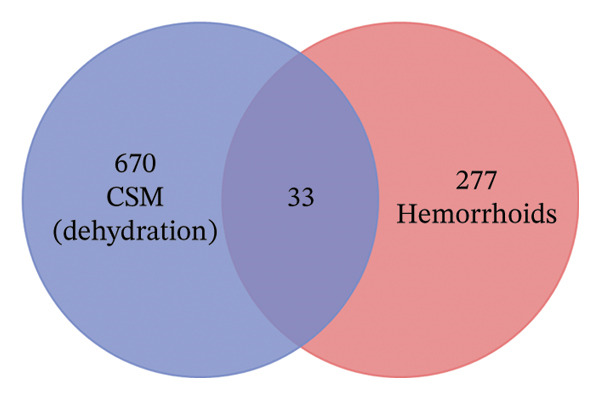
(b)

#### 3.1.3. Construction of a String Network and Screening of Core Target Genes

To construct a string network to predict the strongest gene targets of CSM, the putative gene targets of CSM dried via a hot air oven or dehydration chamber were loaded into Cytoscape. Cytoscape facilitated the identification of the top 5 target genes, as indicated by their degree values, as shown in Figures 2(a) and 2(b). The CSM dried using a hot air oven showed strong genes such as nuclear factor, erythroid 2–like 2 (NFE2L2), NFKB1, Toll‐like receptor 4 (TLR4), and Prostaglandin endoperoxidase synthase 2 (PTGS2). The gene targets of the dehydration chamber−dried CSM were NFE2L2, epidermal growth factor receptor (EGFR), NFKB1, PTGS2, and Arachidonate 5–lipoxygenase (ALOX5). Molecular docking and reverse screening approaches are commonly applied in drug discovery to validate predicted targets and explore structure–activity relationships, thereby supporting mechanism‐based interpretation of network pharmacology results [35, 36].

FIGURE 2Compound−gene interaction of CSM were dried in a hot air oven (a) and in a dehydration chamber (b).

**Figure:**
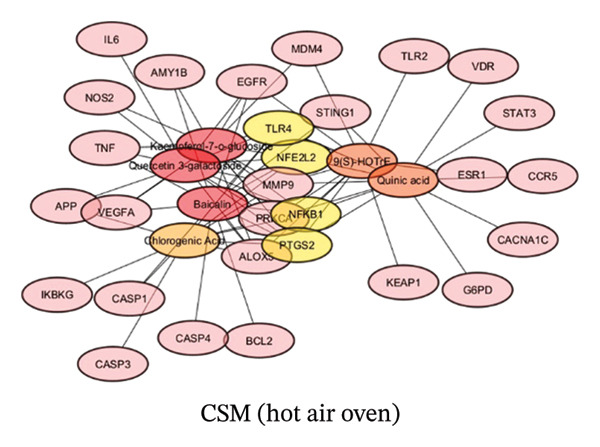
(a)

**Figure:**
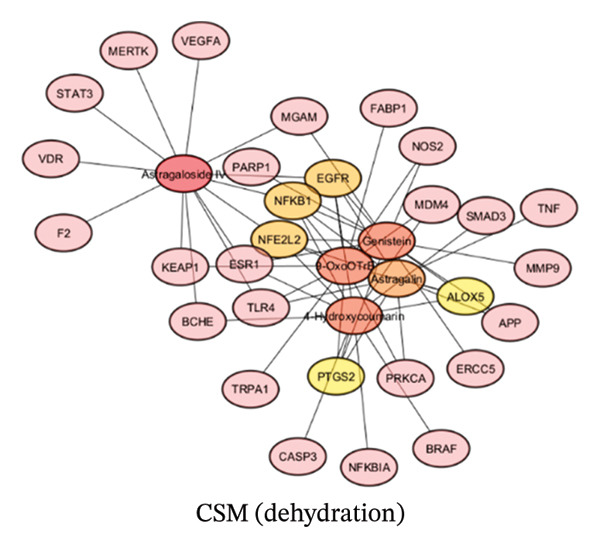
(b)

#### 3.1.4. Cytotoxicity of L929 Cells

**FIGURE 3:**
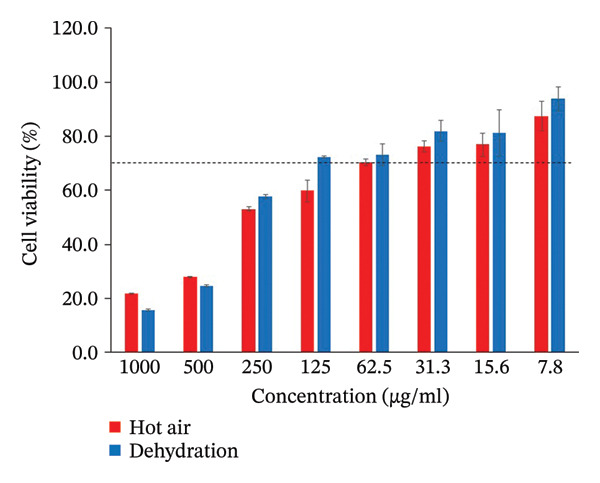
Effect of different drying methods with ethanol extracts of CSM on cell viability of L929 cells. Cells were treated with 7.8–1000 μg/mL of hot air oven and dehydration for 24 h. Cell viability was detected by MTT assay.

The percentage of viable L929 cells after 24 h of exposure to CSM extracts prepared using different drying methods is shown in Figure 3. The dehydration chamber drying method demonstrated the highest safety profile. It was considered safe at concentrations ranging from 7.8 to 125.0 μg/mL. In contrast, hot air drying resulted in lower cell viability, with a safe concentration range limited to 7.8–31.3 μg/mL.

#### 3.1.5. Anti‐Inflammatory Effects and Cytotoxicity in RAW 264.7 Cells

**FIGURE 4:**
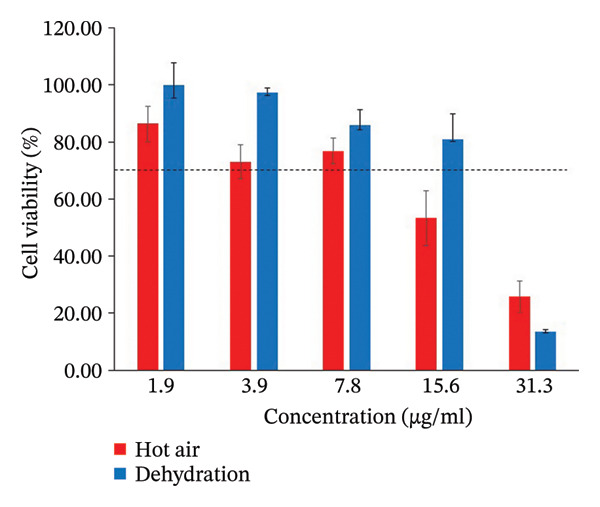
Effect of different drying methods with ethanol extracts of CSM on cell viability and LPS (lipopolysaccharide)‐induced NO (nitric oxide) production in RAW 264.7 cells. Cells were treated with 1.9–31.3 μg/mL of hot air oven and dehydration for 24 h. Cell viability was detected by MTT assay.

**FIGURE 5:**
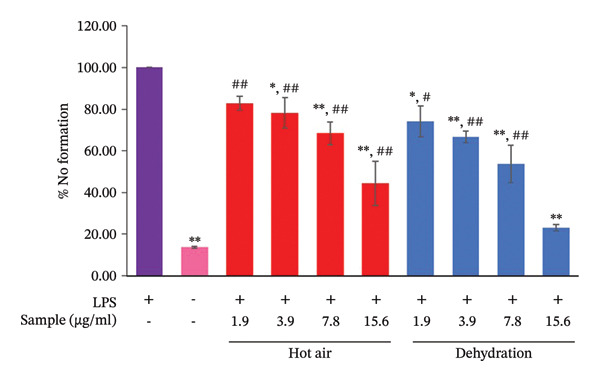
Effects of different drying methods with ethanol extracts of CSM on LPS (lipopolysaccharide)‐induced NO (nitric oxide) production in RAW 264.7 cells. Cells were pretreated with 1.9, 3.9, 7.8, and 15.6 μg/mL of hot air oven and dehydration for 1 h before treatment with 1 μg/mL LPS. After incubation for 24 h, NO production was detected by the Griess test. The values are presented as mean ± SD of four independent experiments. ^∗^ *p* < 0.05 and ^∗∗^ *p* < 0.01, compared to the LPS‐treated group. ^#^ *p* < 0.05 and ^##^ *p* < 0.01, compared to the LPS‐nontreated group.

Anti‐inflammatory efficacy and cytotoxicity were assessed concurrently in the same cell model. As shown in Figure 4, different drying techniques yielded varying levels of cell viability. CSM dried in a hot air oven exhibited the greatest cytotoxicity, with a safe concentration range of 1.9–7.8 μg/mL. In contrast, CSM dried in a dehydration chamber demonstrated a broader safe concentration range of 1.9–15.6 μg/mL. Therefore, the anti‐inflammatory efficacy of CSM, generated via both the hot air oven and the dehydration chamber drying techniques, was evaluated within its respective safe dosage range. CSM dried in the dehydration chamber showed the most potent inhibition of NO production following LPS‐induced inflammation, with an IC_50_ value of 8.50 ± 0.39 μg/mL. Conversely, hot‐air oven drying, which resulted in an IC_50_ of 15.29 ± 3.17 μg/mL for NO inhibition, was deemed unsafe because of significant cytotoxicity at concentrations above 7.8 μg/mL. The observed reduction in %NO for the hot air oven drying method can likely be attributed to decreased cell survival, as the IC_50_ value exceeded the safe concentration range (Figure 5).

#### 3.1.6. Characterization of CSM‐Loaded Niosomes

This approach was based on the hypothesis, supported by previous investigations, that niosomes can improve the delivery and bioavailability of herbal extracts. Ongtanasup et al. [37] reported that putting ginger extract inside niosomes improved drug delivery through the skin and gave them more control over how the drug was released. Moreover, compared to bare ginger extract, ginger‐loaded niosomes have been shown to improve anti‐inflammatory activity [37]. In addition, Nigro et al. [38] reported that compared to the standard drug panthenol, a niosomal formulation of a *Hypericum* Lin extract healed wounds better in mixed‐breed dogs. This was attributed to the increased bioavailability and prolonged therapeutic effects afforded by the niosomal delivery system [38]. Overall, the results show that niosome‐based drug delivery platforms can improve the efficacy of pharmaceutical and herbal medicines while reducing their side effects. Encapsulation of their active compounds within niosomes appears to enhance their pharmacokinetic properties, leading to improved duration and efficacy of action. A solution extracted from CSM dehydrated at a concentration of 180–1000 μg/mL was diluted in 95% ethanol. It was then scanned with a UV spectrophotometer between 200 and 900 nm, as shown in Figure S7. The absorbance of the extracted CSM was measured at 412 nm, and the standard curve was calculated (Figures S8–S10). The CSM‐loaded nanoparticles were then evaluated using Minitab’s estimated conditions.

Residual plots were generated to examine the results of the Box–Behnken experiment and confirm the reliability of the % yield of CSM‐loaded niosomes. As shown in Figure 6(a), the yield of CSM‐loaded niosomes was more significantly impacted by the quadratic terms Span 60, Tween 80, and cholesterol than by the linear or interaction terms, as seen in the Pareto chart (Figure 6(a)), suggesting a nonlinear response pattern. The most significant of these was the quadratic term of cholesterol (*p* = 0.005), which demonstrated its crucial function in controlling the production of vesicles and bilayer stability; too much cholesterol makes membranes more rigid, while too little causes vesicles to collapse [39]. Significant contributions were also made by the quadratic effects of Tween 80 (*p* = 0.025) and Span 60 (*p* = 0.011); however, excessive Tween 80 may compromise vesicle integrity because of its high hydrophilicity, and moderate concentrations of Span 60 promote uniform film formation [40, 41]. In addition, the normal probability plot is shown in Figure 6(b), where points situated close to the referenced red line indicate a normal distribution. In this study, the points were closely aligned with the red line, confirming the normal distribution and validating the model [42]. The residuals vs. fitted values are plotted in Figure 6(c), which suggests that the homoscedasticity of the residuals was assessed across the entire range of predicted values. The random and freeform pattern of the plots indicates that this assumption was reasonably met [43]. Further evidence for assessing normality is provided in Figure 6(d), which illustrates the frequency distribution of the residuals, providing further evidence for assessing normality. The bell‐shaped curve observed in this study indicates a stable working process and normal distribution. The residuals against the order plot in Figure 6(e) were used to identify any discernible patterns that may suggest nonrandomness in the residuals with respect to the sequence of data. The plots showed no apparent pattern in the residuals, indicating that the data were independent of the order of observation [44]. In summary, the analysis of these residual plots confirmed the model’s suitability, as the various assumptions were satisfactorily met.

FIGURE 6The statistical analysis was conducted on the yields of CSM‐loaded niosomes. The yields were shown using a Pareto chart (a). The residual plots were made to look at the results of a Box−Behnken experiment that was used to find out what effects the CSM extract had on niosomes. The normal probability plot (b) assessed the normality of the residuals, with points close to the reference line indicating a strong conformity to the normal distribution. The residuals vs. fitted values plot (c) assessed the residuals’ homoscedasticity across the entire range of projected values. The histogram (d) provided further evidence for assessing normality by illustrating the frequency distribution of the residuals, providing further evidence for assessing normality. The residuals against order plot (e) were used to identify any discernible patterns that may indicate nonrandomness in the residuals with respect to the sequence of data.

**Figure:**
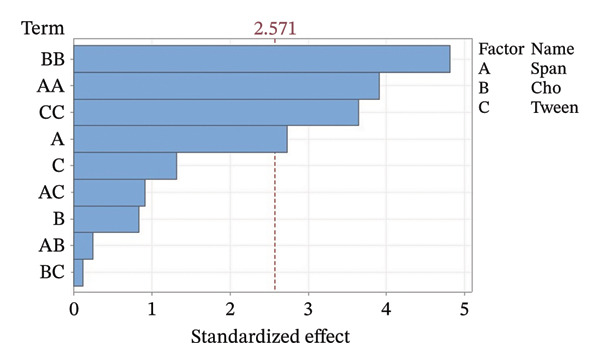
(a)

**Figure:**
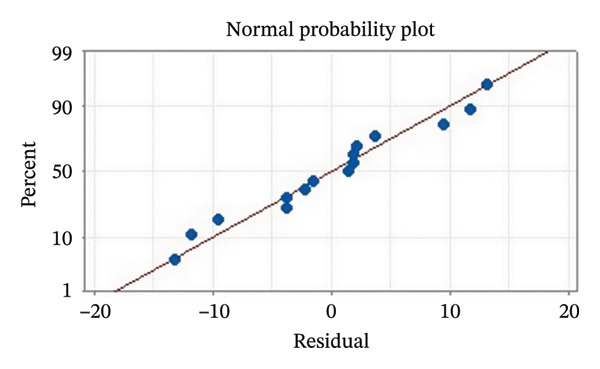
(b)

**Figure:**
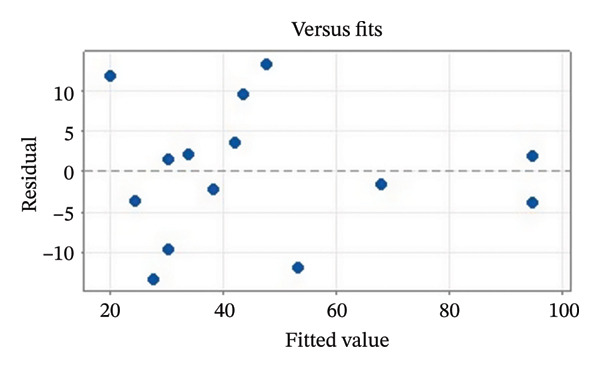
(c)

**Figure:**
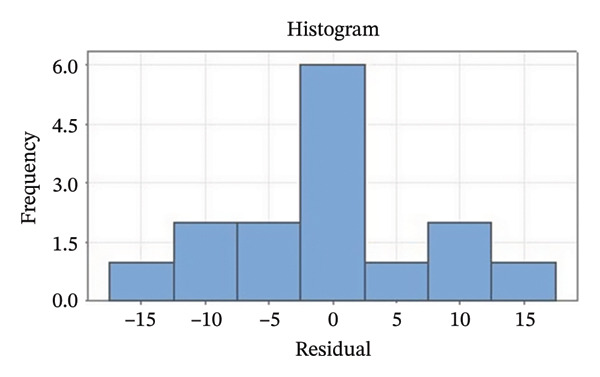
(d)

**Figure:**
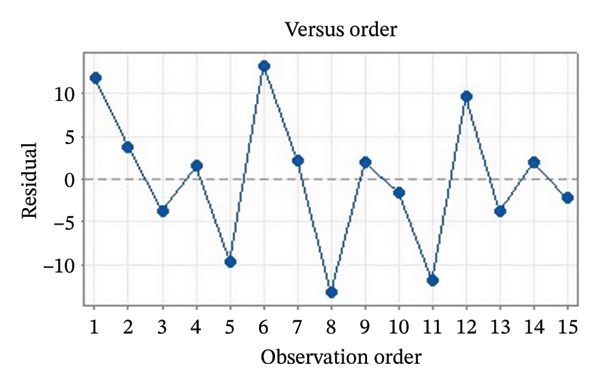
(e)

The Pareto chart in Figure 7(a) reveals that the Span content had the greatest effect on the % EE of the CSM‐loaded niosomes (*p* < 0.05). These findings suggest that the type and amount of surfactant were very important in determining how well the CSM extract was encapsulated in the niosomal formulation. This model worked, as evidenced by the residual plots shown in Figures 7(b), 7(c), 7(d), and 7(e), which indicate that the assumptions were correct. The residuals were spread out normally, had a constant variance, and were not linked to any other residuals. These diagnostic plots revealed that the statistical method used to improve the ability of CSM‐loaded niosomes to be encapsulated was correct. Overall, using Pareto and residual plots to look at both yield andEE gave a full picture of the niosome formulation process. The results highlight important factors, such as the Span content, that significantly influence the performance characteristics of CSM‐loaded niosomes.

FIGURE 7The statistical analysis was conducted on the encapsulation efficiency (% EE) of CSM‐loaded niosomes. The yields were shown using a Pareto chart (a). The residual plots were made to look at the results of a Box−Behnken experiment that was used to find out what effects the CSM extract had on niosomes. The normal probability plot (b) assessed the normality of the residuals, with points close to the reference line indicating a strong conformity to the normal distribution. The residuals vs. fitted values plot (c) assessed the residuals’ homoscedasticity across the entire range of projected values. The histogram (d) provided further evidence for assessing normality by illustrating the frequency distribution of the residuals, providing further evidence for assessing normality. The residuals against order plot (e) were used to identify any discernible patterns that may indicate nonrandomness in the residuals with respect to the sequence of data.

**Figure:**
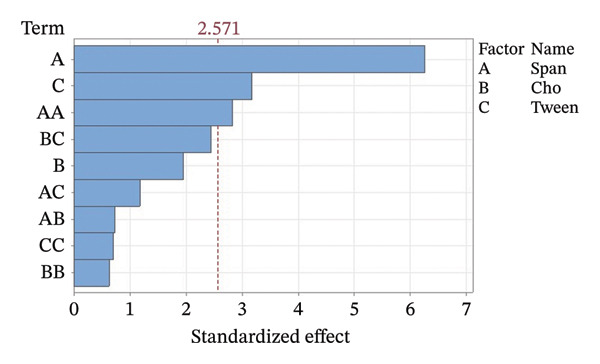
(a)

**Figure:**
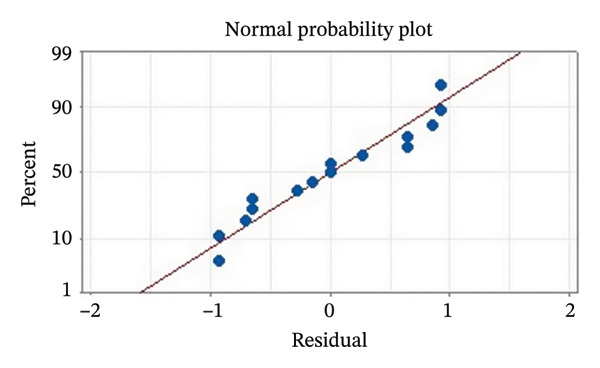
(b)

**Figure:**
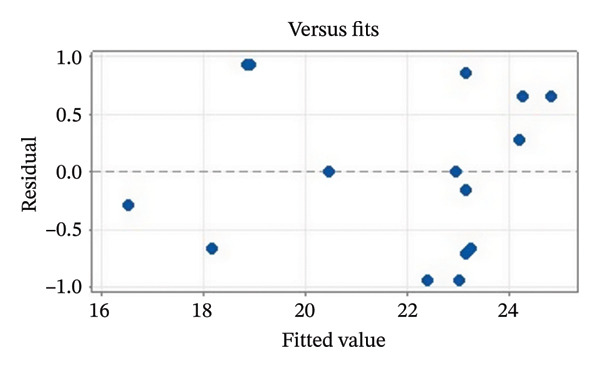
(c)

**Figure:**
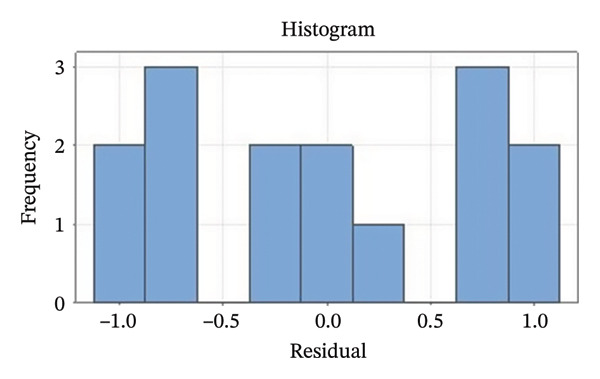
(d)

**Figure:**
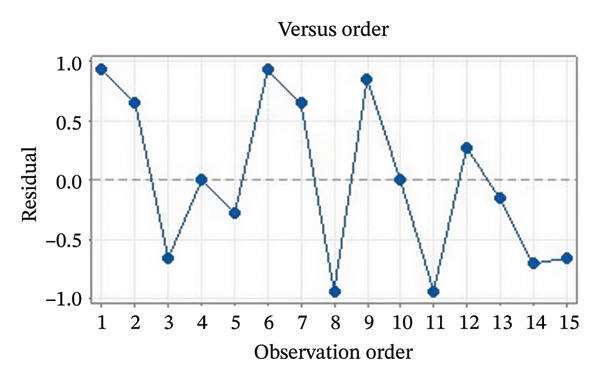
(e)

Table 4 shows the difference ratios between Span 60, Tween 80, and cholesterol, which aided in the determination of the parameter response and regression equation; these values predicted the yield and encapsulation efficiency (% EE), with R‐square values of 91.77% and 93.28%, respectively. The ANOVA results from the response surface regression for yield and % EE are shown in Figures S11 and S12. The contour plot findings revealed the status of CSM‐loaded niosomes. Table 5 shows the measured parameters of CSM‐loaded niosomes, such as the size, charge, yield, and EE. Furthermore, the contour plot revealed correlations of 85.39 ± 6.74 and 57.80 ± 6.95% for the niosome yield and EE (Figure 8), respectively.

**TABLE 4:** Optimized condition of nanoparticles.

Parameter	Charge	
	% yield	% EE
Parameter response	Cholesterol ∗ Cholesterol	Span 60
Confidence level	0.05	0.05
*p* value	0.005	0.002
*R*‐square (%)	91.77	93.28
Regression equation	% Yield = −78.9 + 8.50 Span + 13.58 Cho + 8.73 Tween − 0.476 Span ∗ Span − 0.586 Cho ∗ Cho − 0.443 Tween ∗ Tween + 0.029 Span ∗ Cho + 0.108 Span ∗ Tween + 0.015 Cho ∗ Tween	% EE = 13.83 + 0.356 Span + 0.679 Cho + 0.520 Tween − 0.0301 Span ∗ Span − 0.0067 Cho ∗ Cho − 0.0075 Tween ∗ Tween − 0.0075 Span ∗ Cho + 0.0120 Span ∗ Tween − 0.0250 Cho ∗ Tween

*Note:* *p* values < 0.05 indicate statistically significant model terms. Cholesterol^2^ (*p* = 0.005) was the most influential factor for % yield, while Span 60 is significantly affected by % EE (*p* = 0.002). Both models were statistically significant (*p* < 0.05).

**TABLE 5:** The characterization of the optimum condition of CSM‐loaded niosomes.

Parameter	CSM‐loaded niosome
Size (nm)	208.76 ± 20.28
PDI	0.51 ± 0.04
Charge (mV)	30.10 ± 1.75
% Yield	85.39 ± 6.74
% EE	57.80 ± 6.95

FIGURE 8The contour plot of yield and encapsulation efficiency (% EE) of CSM‐loaded niosomes.

**Figure:**
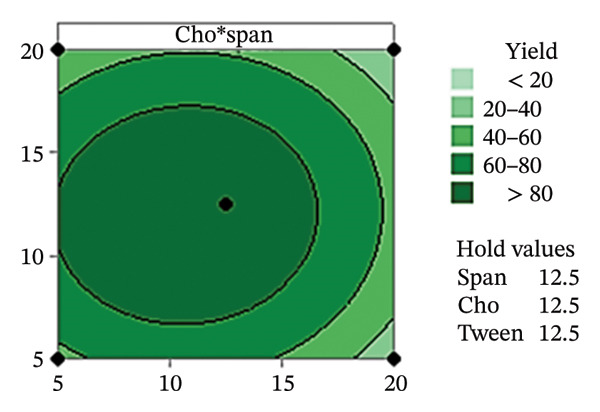
(a)

**Figure:**
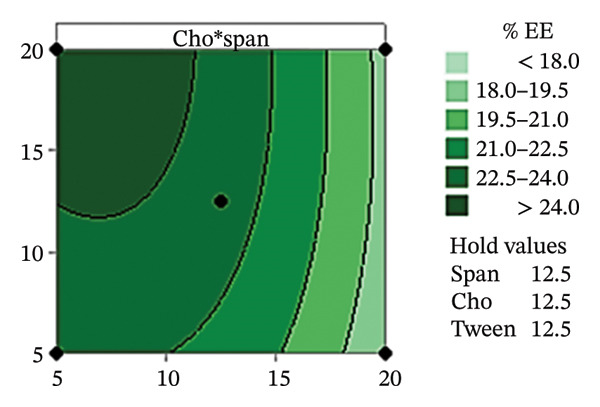
(b)

Differences in the drying process significantly impact yield, with temperature being a key factor [45].

The % yield of the extract, bioactive compounds, aromatic oils, and alterations in cellular structure are reduced because of the high temperature during the dying process [46]. Furthermore, antioxidant activity decreases substantially when drying temperatures exceed 40°C [47, 48]. Notably, shade drying preserves essential oils and bioactive compounds and is second only to freeze‐drying in terms of efficacy [49, 50]. Quinic acid was identified as the primary active compound. Quinic acid has demonstrated potent anti‐inflammatory properties in various studies. It inhibits the MAP kinase and NF‐κB pathways and reduces TNF‐α stimulation in vascular smooth muscle cells by suppressing VCAM‐1 expression [51, 52]. In addition, dicaffeoylquinic acid has been shown to inhibit NO production in LPS‐treated RAW 264.7 macrophages at an effective dose of 10 μM [53]. The major constituents of dehydration chamber−dried CSM are flavonoids such as astragalin and genistein, the saponin Astragaloside IV, the long‐chain fatty acid 9‐OxoOTrE, and the phenolic acid 4‐hydroxycoumarin. A literature review revealed that astragalin has anti‐inflammatory effects through the regulation of the NF‐κB and MAPK signaling pathways [54, 55]. By inhibiting NF‐κB, PGs, iNOS, cytokines, and ROS, genistein has been shown to have strong anti‐inflammatory effects [56]. 4‐Hydroxycoumarin has been shown to have potent anti‐inflammatory activity through the inhibition of COX‐II enzyme activity in Sprague−Dawley rats and in the treatment of ulcerative colitis at 25 mg/kg in rats by decreasing the loss of glutathione in the context of colonic inflammation [57, 58]. However, anti‐inflammatory information for 9‐OxoOTrE is lacking. On the basis of the LC‐MS analysis of the active compound content, the key active compound in the CSM samples dried using the dehydration chamber method was 4‐hydroxycoumarin.

Bioinformatics analysis of compounds in CSM processed by hot air oven drying revealed significant compound‒gene interactions relevant to hemorrhoids: Kaempferol‐7‐o‐glucoside emerged as a key compound and exhibited the strongest binding affinity to hemorrhoid‐related genes. The 14 genes it interacted with were NOS2, ALOX5, EGFR, AMY1B, NFE2L2, TLR4, TNF, NFKB1, PTGS2, VEGFA, PRKCA, IL‐6, MDM4, and MMP9. Recent studies have reported its diverse therapeutic potential, encompassing anti‐inflammatory effects through the inhibition of proinflammatory molecules [59], cytoprotective properties, anticancer activities, and even anti‐HIV‐1 efficacy [60, 61]. Thirteen genes were shown to interact with quercetin 3‐galactoside: NOS2, ALOX5, EGFR, NFE2L2, TLR4, TNF, PTGS2, NFKB1, VEGFA, PRKCA, IL‐6, APP, and MMP9. As a strong antioxidant [62] and by blocking the Gal‐3‐NLRP3 signaling pathway [63], it can change atherosclerotic inflammation. A hydroxy‐octadecatrienoic acid derivative called 9(S)‐HOTTE interacted with 13 genes, such as ALOX5, EGFR, BCL2, NFE2L2, TLR4, PTGS2, NFKB1, ESR1, TLR2, KEAP1, and PRKCA. Sun et al. [64] reported its efficacy in reducing hepatic and muscular inflammation. Quinic acid, the major compound in CSM processed by a hot air oven, is bound to 12 genes, namely, VDR, CACNA1C, G6PD, NFE2L2, TLR4, STAT3, CCR5, PTGS2, STING1, NFKB1, PRKCA, and MMP9. Multiple studies have shown robust anti‐inflammatory properties, including a reduction in TNF‐α levels in vascular smooth muscle cells via inhibition of VCAM‐1 expression and decreased NO production in LPS‐treated RAW 264.7 cells [51–53]. Baicalin was reported by Dacrema et al. [65] to have potent antioxidant, anti‐inflammatory, and neuroprotective effects. Moreover, it has been shown to benefit patients with pulmonary diseases and hypertension, key to its role in adjective cognitive function and its ability to relieve anxiety and depression symptoms, Liu et al. [66]. Our study revealed that baicalin interacts with 12 genes, namely, ALOX5, EGFR, AMY1B, NFE2L2, TLR4, TNF, CASP1, PTGS2, STING1, NFKB1, VEGFA, and CASP3. It has been reported that CGA, which is a polyphenolic compound, has strong antioxidant activity, which can be related to reducing the effects of inflammation on diabetes and heart diseases [67].

Astragaloside IV is an active compound in the CSM dehydration process and has the highest binding affinity for 14 genes associated with hemorrhoids, including VDR, MGAM, EGFR, BCHE, MERTK, NFE2L2, TLR4, STAT3, F2, NFKB1, ESR1, VEGFA, and KEAP1. Antioxidative stress, anti‐inflammatory, and wound healing activities have been reported for Astragaloside IV [68, 69] and are related to the management of cardiovascular diseases [70]. 4‐Hydroxycoumarin, the most abundant compound in the CSM postdehydration chamber drying, exhibited binding interactions with 12 genes, namely, ALOX5, SMAD3, FABP1, EGFR, BCHE, NFE2L2, PTGS2, NFKB1, ESR1, NFKBIA, BRAF, MDM4, and CASP3. Astragaloside IV has been shown to have anti‐inflammatory effects through the suppression of NF‐kB and MAPK activities [71]. In addition, 4‐hydroxycoumarin has been shown to be an anticancer agent and to have potent antimicrobial properties [72, 73]. This study presents novel findings regarding 9‐OxoOTrE, a long‐chain fatty acid, and its potential antihemorrhoidal properties. 9‐OxoOTrE was found to interact with 12 genes, namely, NOS2, ALOX5, FABP1, EGFR, NFE2L2, TLR4, TRPA1, PTGS2, NFKB1, KEAP1, PRKCA, and MDM4. Astragalin, or kaempferol‐3‐o‐glucoside, has been reported to have antioxidant and inflammatory effects [74]. Astragalin has been reported to benefit cardiovascular health by improving endothelial function and reducing blood pressure [75]. Our analysis revealed that astragalin binds 12 genes, including NOS2, ALOX5, EGFR, NFE2L2, TLR4, TNF, PTGS2, NFKB1, PRKCA, APP, and MMP9. Genistein is a type of isoflavone that binds to 11 genes, namely, ERCC5, MGAM, ALOX5, EGFR, NFE2L2, PTGS2, NFKB1, ESR1, APP, MMP9, and PARP1. In 2023, the role of genistein in reducing the risk of chronic diseases such as cancer and cardiovascular conditions was determined [76]. The anti‐inflammatory effects of genistein may be involved in inflammation‐related diseases [77]. Moreover, the use of genistein as a phytoextrogenic agent is potentially beneficial for improving menopausal symptoms related to reducing hormone‐related cancer [78]. In conclusion, a literature review of compounds in both CSM preparations consistently revealed anti‐inflammatory properties, supporting the potential of CSM as an anti‐inflammatory agent.

The dehydration chamber drying method demonstrated the highest safety profile, with cell viability exceeding 70% (the threshold for nontoxicity according to ISO 10993‐5; [30]). The MTT assay was chosen for the assessment of cellular viability owing to its cost‐effectiveness, precision, appropriate duration, and high reproducibility [79, 80]; this colorimetric assay uses a spectrophotometer to measure the ability of live mitochondria to convert yellow MTT into purple formazan.

In this study, inflammation was induced by LPS to stimulate reactive oxygen species (ROS) production and oxidative stress and to bind to TLR4 on immune surfaces. This activates the NF‐κB and MAPK pathways to produce inflammatory cytokines and ultimately apoptosis [81]. Moreover, NF‐κB activation upregulates iNOS expression, resulting in increased NO production [82]. Consequently, NO serves as a key marker for assessing inflammatory activity. Our anti‐inflammatory assays revealed that CSM dried using a hot air oven lacked anti‐inflammatory efficacy, as its IC_50_ for NO reduction exceeded the safe dosage range. Conversely, CSM dried in a dehydration chamber demonstrated anti‐inflammatory activity at lower, nontoxic doses. Several compounds in dehydrated CSM, such as astragalin, genistein, Astragaloside IV, 9‐OxoOTrE, and 4‐hydroxycoumarin, are likely to work together to cause this effect. These chemicals are related to important regulatory genes such as NFE2L2, EGFR, NFKB1, PTGS2, and ALOX5, which play a role in controlling inflammation. The nuclear factor kappa B subunit 1 (NF‐κB1) gene plays an important role in regulating inflammatory responses. When stimulated, NF‐κB increases the expression of various proinflammatory cytokines, including IL‐8, IL‐1B, IL‐6, and TNF‐α [83–86]. Excessive NF‐κB expression can lead to chronic inflammation. In addition, the expression of TLR4, a gene that enhances NF‐κB activity, is induced by ROS and oxidative stress [81, 87]. While the EGFR is not directly involved in inflammation, it can modulate the inflammatory response by activating the production of proinflammatory cytokines, key initiators of inflammation. Previous research has demonstrated that EGFR phosphorylation is essential for TLR4‐mediated macrophage activation during sepsis [88]. In the infectious state, EGFR can also lead to apoptosis of CD4+ T lymphocytes by promoting the Warburg effect through the TBK1/Glut1 signaling pathway [89]. COX2/PTGS2 is an early response gene that stimulates inflammation, increasing prostaglandin synthesis and inducing the expression of other proinflammatory genes [90]. ALOX5 synthesizes leukotriene B4 and cysteinyl leukotrienes from arachidonic acid. These leukotrienes increase neutrophil recruitment in the context of inflammation and facilitate recovery from acute inflammation [91]. Conversely, NFE2L2 is a master regulator that prevents inflammation caused by antioxidative stress [92]. Therefore, active compounds capable of inhibiting NF‐κB, NFKB1, TLR4, PTGS2, and EGFR expression while promoting NFE2L2 expression may be potent anti‐inflammatory agents. The CSM generated via the two drying methods had some affected genes in common, namely, NFKB1, PTGS2, and NFE2L2. Nevertheless, dehydrated CSM has different effects on EGFR and ALOX5 [88, 91]. These findings suggest that EGFR and ALOX5 may play crucial roles in enhancing the anti‐inflammatory activity of CSM dried in a dehydration chamber. Using string network analysis, the anti‐inflammatory mechanism of CSM dehydration was further elucidated. The analysis revealed that compounds within dehydrated CSM could inhibit NF‐κB1, PTGS2, ALOX5, and EGFR expression while promoting NFE2L2 expression.

The aim of this study was to increase the delivery of a compound extracted from CSM by encapsulating it within niosomes. This approach was dependent on the hypothesis, supported by previous investigations, that niosomes can improve the delivery and bioavailability of herbal extracts. It has been reported that putting ginger extract inside niosomes improved drug delivery through the skin and allowed for more control over how the drug was released [37]. Moreover, compared to bare ginger extract, ginger‐loaded niosomes showed increased anti‐inflammatory activity [37]. Moreover, Nigro et al. [38] reported that a niosomal formulation of a *Hypericum* Lin extract healed wounds better in mixed‐breed dogs than the standard drug, panthenol. This finding supports the increased bioavailability and prolonged therapeutic capability afforded by the niosomal delivery system [38]. Overall, the results show that niosome‐based drug delivery platforms can improve the efficacy of pharmaceutical and herbal medicines while reducing their side effects. The encapsulation of compounds within niosomes appears to increase their pharmacokinetic properties, leading to improved efficacy and duration of action. A solution extracted from dehydrated CSM at a concentration of 180–1000 μg/mL was diluted in 95% ethanol prior to scanning with a UV spectrophotometer between 200 and 900 nm. The wavelength of the extracted CSM was measured at 412 nm, and the standard curve was calculated. The CSM‐loaded nanoparticles were then created using Minitab’s estimated conditions. Furthermore, the cholesterol content was the major characteristic that influenced yield. The presence of cholesterol contributes to the stability of the niosome membrane, reducing fluid loss and permeability by enhancing hydrogen bond interactions [93]. In addition, the cholesterol content can increase the area per molecule; therefore, CSM−niosome loading results in a large unilamellar vesicle (LUV) with a diameter between 100 and 3000 nm [94, 95]. The Span 60 content was the parameter that affected % EE the most. These results align with those of previous studies that identified Span 60 as a crucial factor in % EE. This is due to the structure of Span 60, which possesses two polar properties: hydrophilic and hydrophobic. As a result, low‐solubility CSM can be trapped by enhancing the bilayer arrangement [96]. Furthermore, the contour plot revealed the conditions that led to yields and EE levels exceeding 80% and 24%, respectively. The optimal ratio of Span 60:Tween 80:cholesterol of 11.0:19.0:13.5 is proposed.

## 4. Conclusions

This research emphasizes the importance of drying CSM extracts in a dehydration chamber, as it significantly aids in the retention of its therapeutic value in hemorrhoid treatment. Drying in a dehydration chamber greatly retains bioactive components as opposed to drying using a hot air oven. It is critical to note the changes in the regulatory genes in dehydrated CSM, such as EGFR and ALOX5, which enhance anti‐inflammatory properties, as they help alleviate the inflammation, pain, and irritation associated with hemorrhoids. In addition, this study highlights the first findings concerning the long‐chain fatty acid 9‐OxoOTrE, which is antihemorrhoidal, and its possible antihemorrhoidal activities with respect to CSM dehydration. Furthermore, this study presents formulations of CSM−niosomes with optimized parameters. According to the optimization results, all three formulation components, Span 60, Tween 80, and cholesterol, significantly affected the yield and encapsulation effectiveness of the niosomes. Among all the components, cholesterol displayed the greatest quadratic effect in terms of yield, which suggests its importance in the stability of the bilayer membrane and its control of vesicle formation. Low and moderate concentrations of Span 60 improve vesicle integrity, whereas excessive concentrations result in low yield because of low dispersion caused by high viscosity.

The optimum ratio in the case of Span 60: Tween 80: cholesterol was 11.0:19.0:13.5. This dosage form is capable of improving the treatment of hemorrhoids through more effective targeting of active compounds to inflamed regions, as well as their sustained retention, which enhances the relief of inflammatory symptoms in a more effective and long‐lasting manner. The findings not only advance the understanding of the possible applications of CSM but also pave the way for the creation of new hemorrhoid treatments and other anti‐inflammatory plant‐based therapies. The future underpinning of these findings could optimize existing hemorrhoid management treatment through inflammation, pain, and edema control, which ultimately enhances patients’ quality of life. To achieve localized delivery and systemic sparing, the current investigation was aimed at topical niosomal delivery for hemorrhoid management of CSM‐derived composites for the sake of improved axial retention equilibrium.

## Author Contributions

Anunya Suksanga: conceptualization, methodology, investigation, data curation, formal analysis, visualization, and writing−original draft.

Komgrit Eawsakul: conceptualization, methodology, resources, investigation, data curation, formal analysis, validation, formal analysis, and writing−review and editing.

Kingkan Bunluepuech: conceptualization, methodology, resources, investigation, data curation, formal analysis, visualization, supervision, project administration, funding acquisition, and writing−review and editing.

## Funding

This work was supported by Walailak University Research Assistant (Grant No. WU 05).

## Conflicts of Interest

The authors declare no conflicts of interest.

## Supporting Information

Supporting information related to this article are provided as a separate file, including Supporting Figures S1–S15 showing chromatograms, compound structures, UV–Vis spectra, response surface analysis, and MS/MS spectra, as well as Supporting Tables S1–S5 presenting the LC‐MS−identified compounds and MS/MS fragmentation characteristics of compounds detected in CSM extracts.

## Supporting information


**Supporting Information** Additional supporting information can be found online in the Supporting Information section.

## Data Availability

The data used to support the findings of this study are available from the corresponding author upon request.
