# Supplementary material for: Preparation and Evaluation of Niosomal Clerodendrum serratum (Linn.) Moon Extract Formulations: Comparative In Silico and In Vitro Studies of Drying Methods for the Treatment of Hemorrhoids
Source: Scientifica (Cairo). 2026 Apr 29;2026:3662572. doi: 10.1155/sci5/3662572 (PMC13126093; doi:10.1155/sci5/3662572)
Supplement: Supplementary file 1 — Supporting Information Additional supporting information can be found online in the Supporting Information section. [file SCI5-2026-3662572-s001.zip › SuppFig12.pptx]

## Slide 1
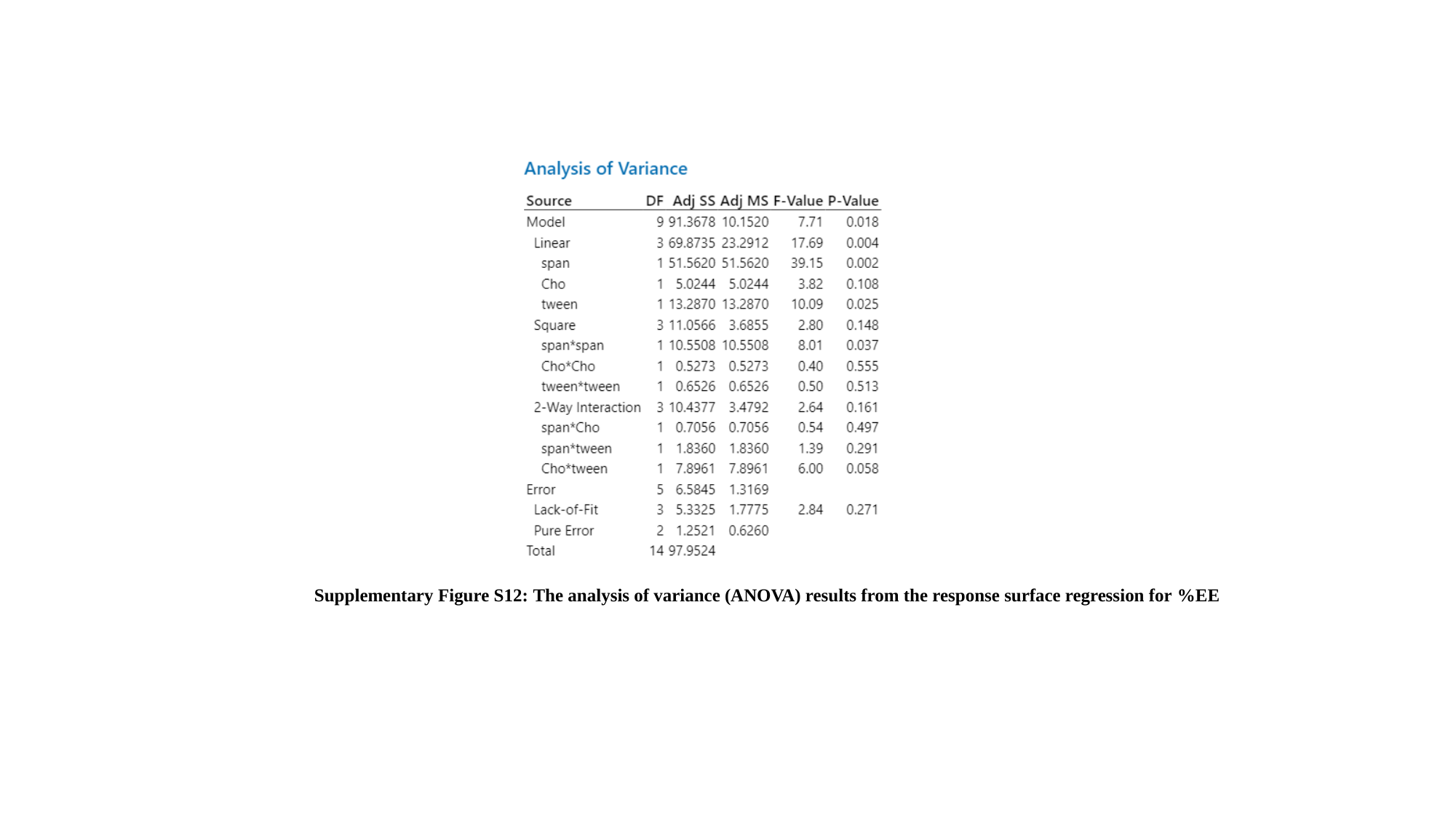

Supplementary Figure S12: The analysis of variance (ANOVA) results from the response surface regression for %EE
