# Supplementary material for: Preparation and Evaluation of Niosomal Clerodendrum serratum (Linn.) Moon Extract Formulations: Comparative In Silico and In Vitro Studies of Drying Methods for the Treatment of Hemorrhoids
Source: Scientifica (Cairo). 2026 Apr 29;2026:3662572. doi: 10.1155/sci5/3662572 (PMC13126093; doi:10.1155/sci5/3662572)
Supplement: Supplementary file 1 — Supporting Information Additional supporting information can be found online in the Supporting Information section. [file SCI5-2026-3662572-s001.zip › Supplementary data.docx]

**Supplementary data**

Preparation and Evaluation of Niosomal *Clerodendrum serratum* (Linn.) Moon Extract Formulations: A Comparative Study of Drying Methods for the Treatment of Hemorrhoids by in silico and in vitro studies

Anunya Suksanga^1^, Komgrit Eawsakul^1, 2^, Kingkan Bunluepuech^1,3^*

^1^ Research Excellence Center for Innovation and Health Products (RECIHP), Walailak University, Nakhon Si Thammarat 80160, Thailand

^2^ Department of Applied Thai Traditional Medicine, School of Medicine, Walailak University, Nakhon Si Thammarat 80160, Thailand

^3^ School of Allied Health Sciences*,* Walailak University, Nakhon Si Thammarat 80160, Thailand

Correspondence should be addressed to Kingkan Bunluepuech; kingkan.bu@wu.ac.th

Corresponding Authors:

Kingkan Bunluepuech

School of Allied Health Sciences, Walailak University, Nakhon Si Thammarat 80160, Thailand

Email address: kingkan.bu@wu.ac.th

**Supplementary Figure S1: Chromatogram in + ESI scan mode of the *Clerodendrum serratum* (Linn.) Moon (CSM) drying with hot air oven via, showing the major retention times of detected compounds.**


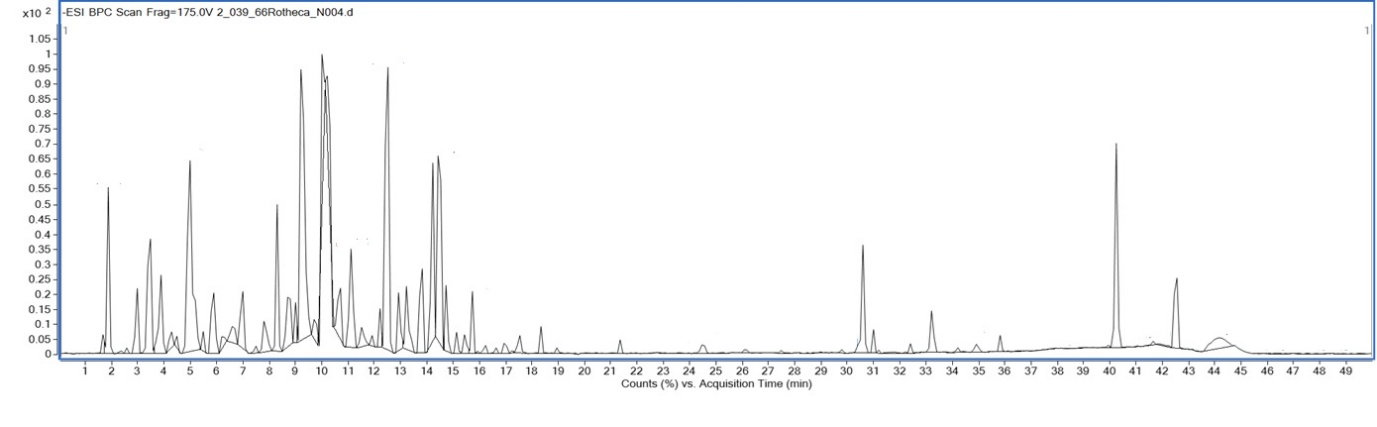


**Supplementary Figure S2: Chromatogram in - ESI scan mode of the *Clerodendrum serratum* (Linn.) Moon (CSM) drying with hot air oven via, showing the major retention times of detected compounds.**

**Supplementary Figure S3:** **Chromatogram in + ESI scan mode of the *Clerodendrum serratum* (Linn.) Moon (CSM) drying with dehydration chamber, showing the major retention times of detected compounds.**

**Supplementary Figure S4: Chromatogram in - ESI scan mode of the *Clerodendrum serratum* (Linn.) Moon (CSM) drying with dehydration chamber, showing the major retention times of detected compounds.**


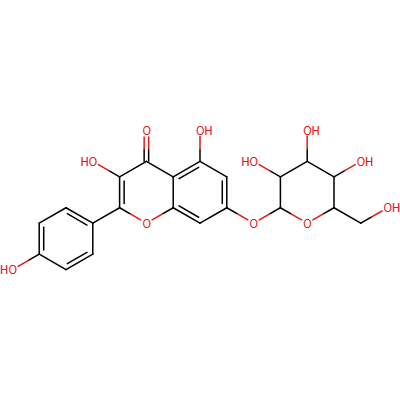


**Supplementary Figure S5**: **Sup Kaempferol-7-O-glucoside structure**

(Web tools used: <https://www.rcsb.org/chemical-sketch> , Access on 4 October 2025)


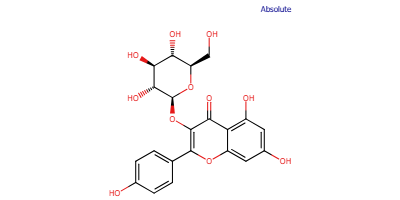


**Supplementary Figure S6: Astragalin structure**

(Web tools used: <https://www.rcsb.org/chemical-sketch> , Access on 4 October 2025)


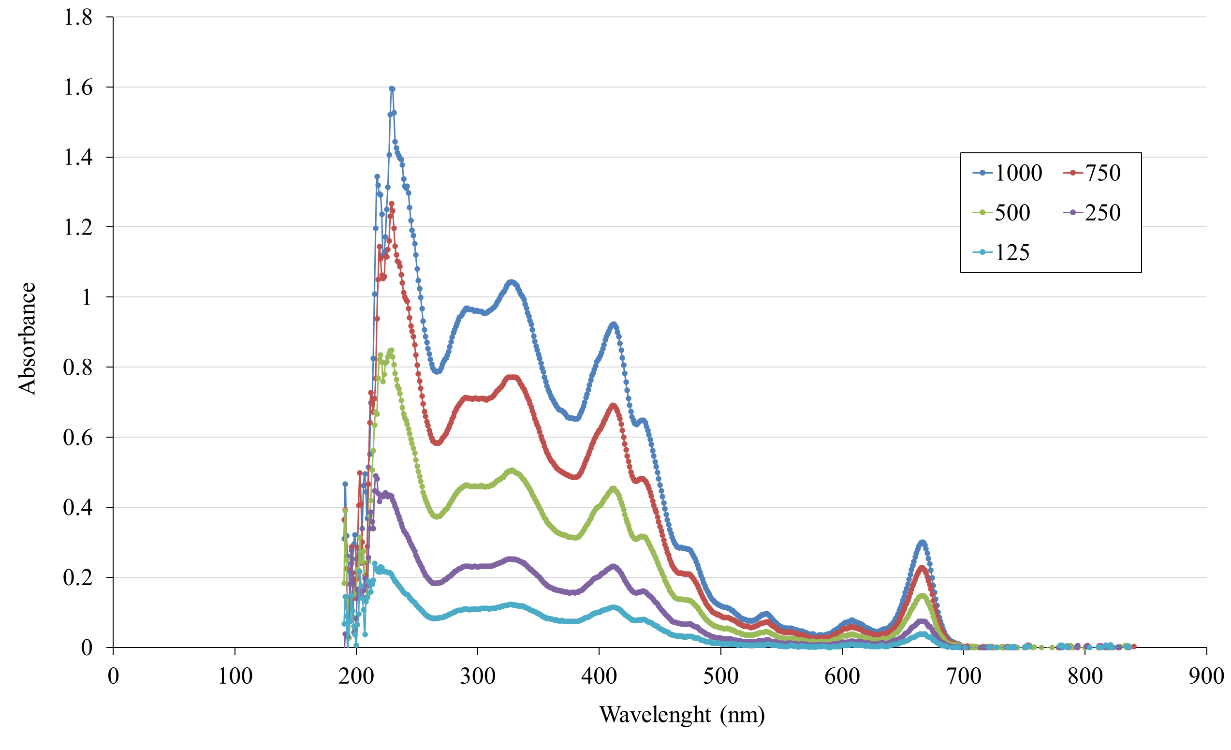


**Supplementary Figure S7: The CSM extract scanned over the wavelength range between 200–900 nm**

**
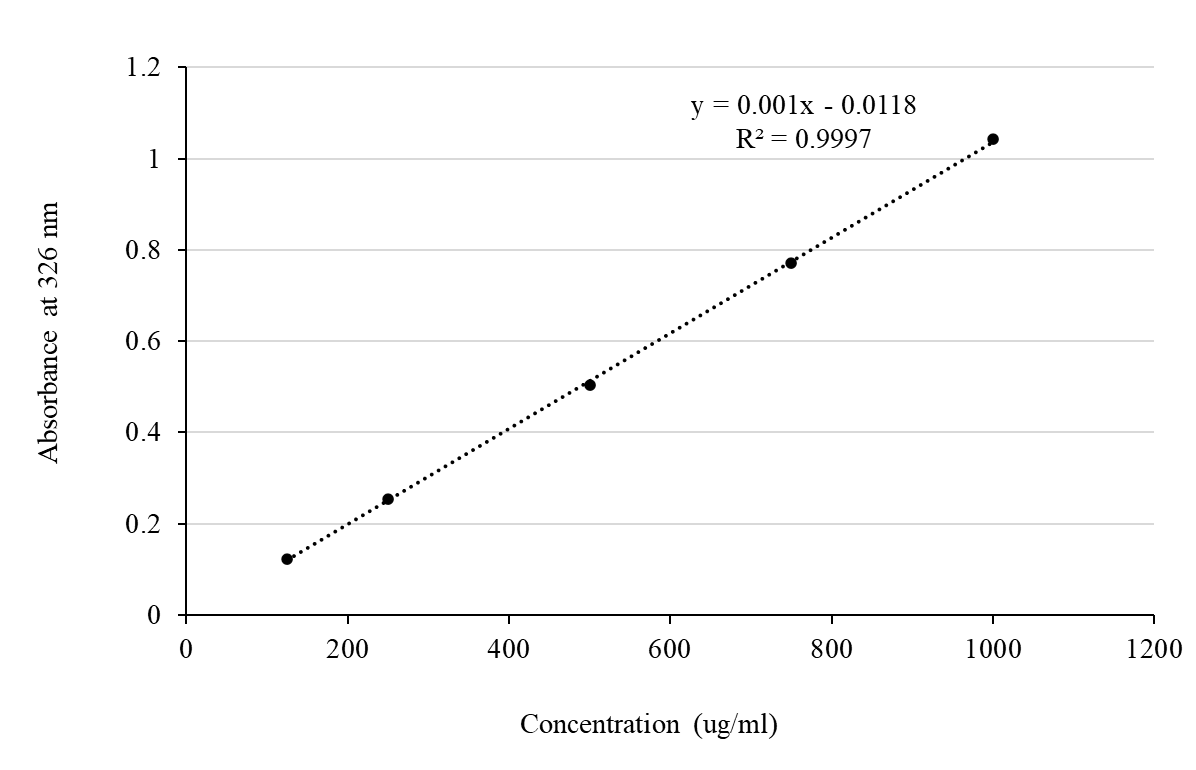
**

**Supplementary Figure S8: The standard curve of CSM extract scanned over the wavelength 326 nm**


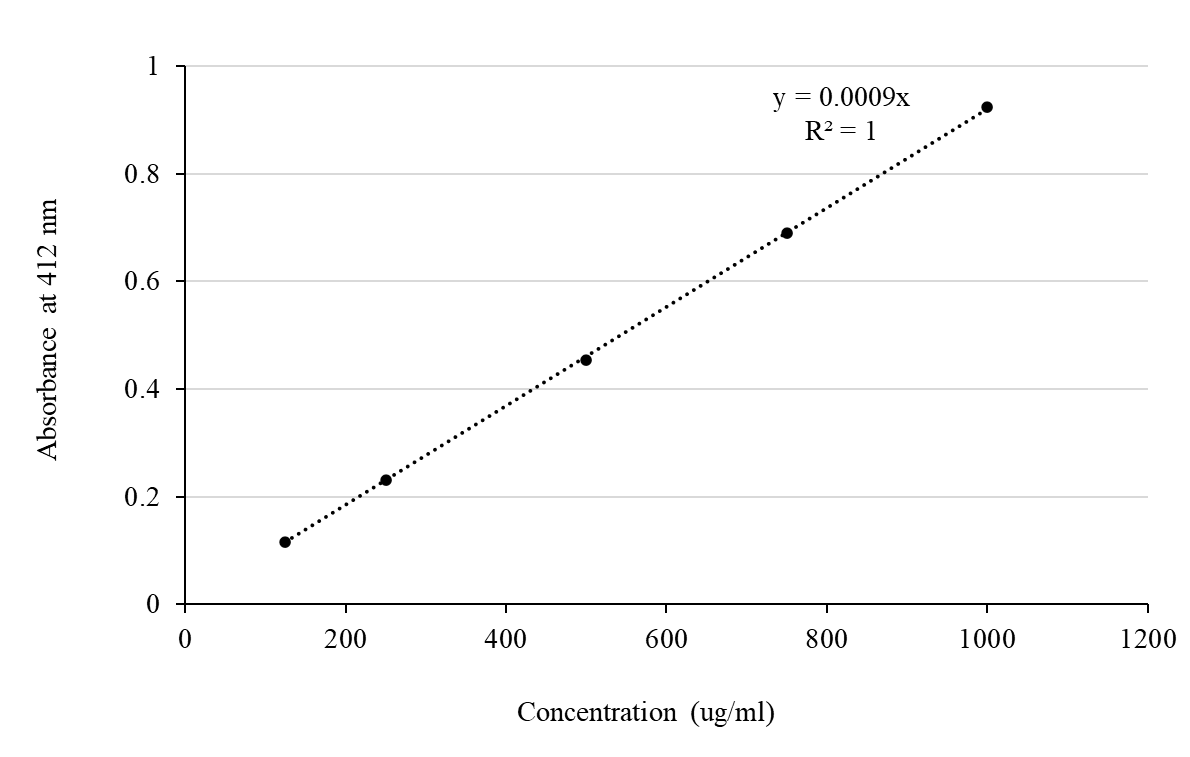


**Supplementary Figure S9: The standard curve of CSM extract scanned over the wavelength 412 nm**

**
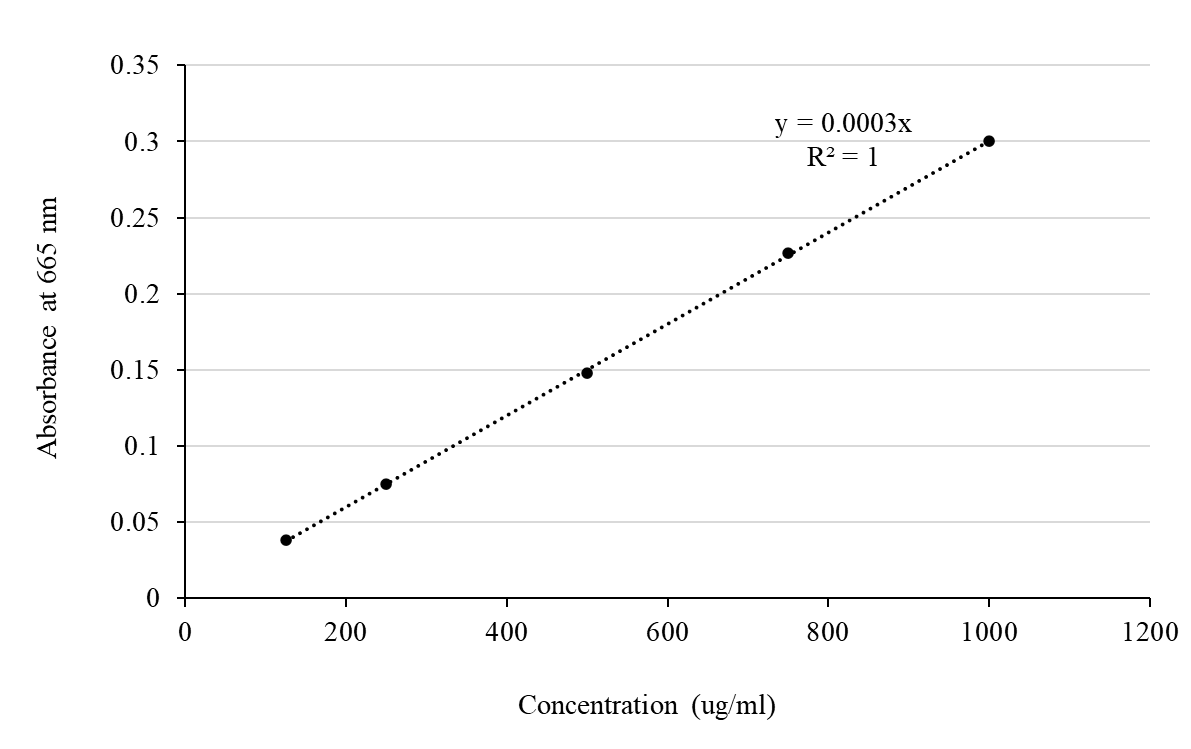
**

**Supplementary Figure S10: The standard curve of CSM extract scanned over the wavelength 665 nm**


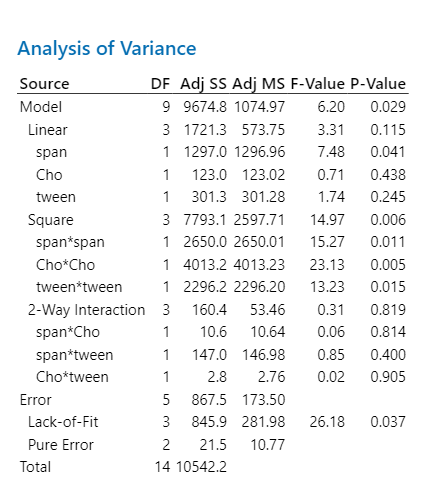


**Supplementary Figure S11: The analysis of variance (ANOVA) results from the response surface regression for yield**


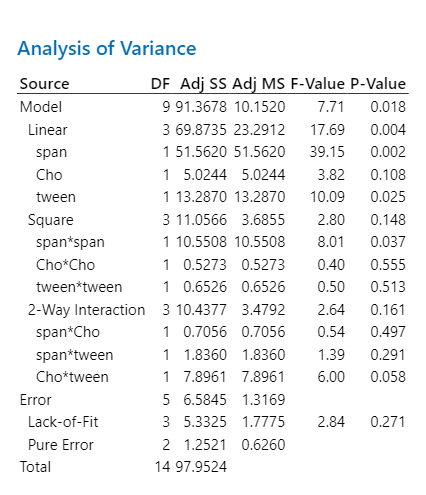


**Supplementary Figure S12: The analysis of variance (ANOVA) results from the response surface regression for %EE**


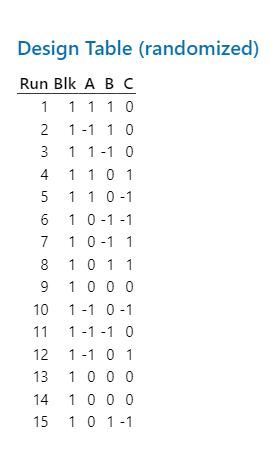


**Supplementary Figure S13:** **The Box–Behnken design for optimized condition of nanoparticles.**


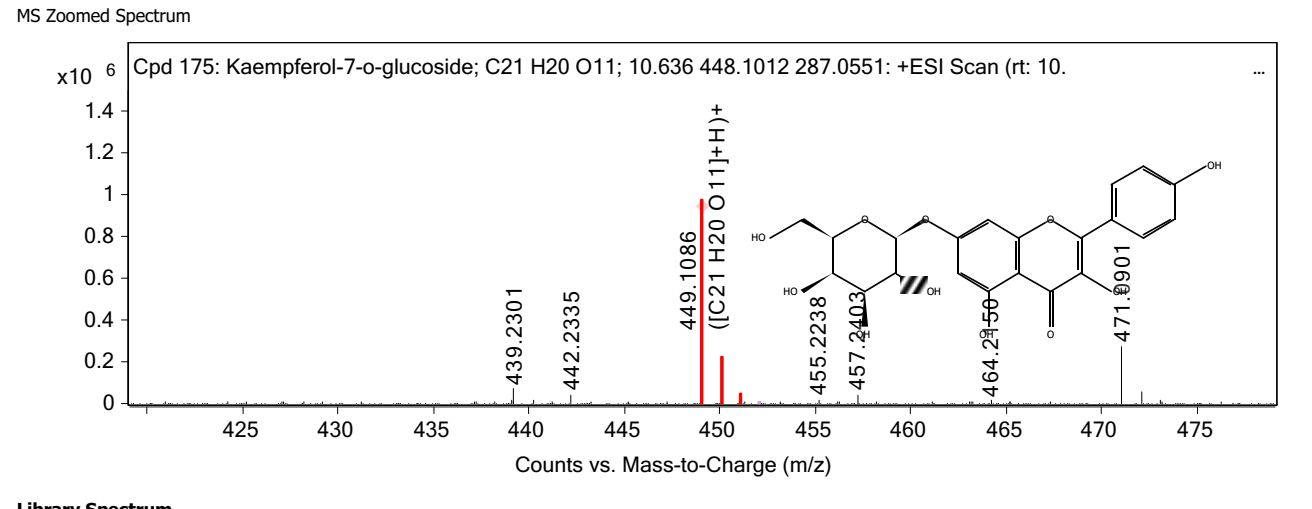


**Supplementary Figure S14. MS/MS spectrum of kaempferol-7-O-glucoside exhibiting a similar fragmentation pattern consistent with flavonoid glycosides.**


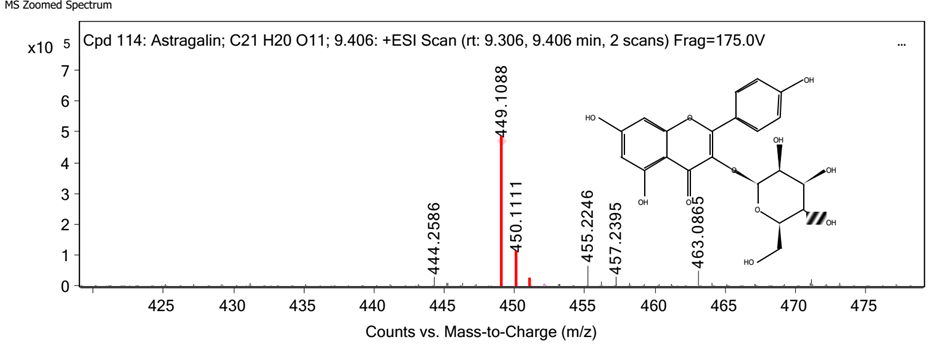


**Supplementary Figure S15. MS/MS spectrum of Astragalin (kaempferol-3-O-glucoside) showing characteristic fragment ions derived from glycosidic bond cleavage.**

**Supplementary Table S1 Compounds identified in the *Clerodendrum serratum* (Linn.) Moon (CSM) drying with hot air oven via positive electrospray ionization (ESI+) LC-MS analysis**

| No. | m/z | RT (min) | Name of the compound | Molecular Formulae | Molecular weight | %  Con  tent | Matching  Score | Diff (DB, ppm) |
| --- | --- | --- | --- | --- | --- | --- | --- | --- |
| 1 | 449.1083 | 9.405 | Kaempferol-7-o-glucoside | C21 H20 O11 | 448.1011 | 1.07 | 95.68 | -1.12 |
| 2 | 447.0929 | 11.175 | Baicalin | C21 H18 O11 | 446.0857 | 1.35 | 95.90 | -1.66 |
| 3 | 343.2255 | 19.725 | 16R-HETE | C20 H32 O3 | 320.2361 | 0.62 | 91.89 | -2.88 |
| 4 | 277.2171 | 30.546 | Stearidonic Acid | C18 H28 O2 | 276.2098 | 0.58 | 90.37 | -3.1 |

**Supplementary Table S2 Compounds identified in the *Clerodendrum serratum* (Linn.) Moon (CSM) drying with hot air oven via positive electrospray ionization (ESI-) LC-MS analysis**

| No. | m/z | RT (min) | Name of the compound | Molecular Formulae | Molecular weight | %  Con  tent | Matching  Score | Diff (DB, ppm) |
| --- | --- | --- | --- | --- | --- | --- | --- | --- |
| 1 | 503.1616 | 1.82 | Raffinose | C18 H32 O16 | 504.1691 | 0.26 | 92.60 | -0.14 |
| 2 | 179.0562 | 1.845 | Allose | C6 H12 O6 | 180.0635 | 0.02 | 90.0 | -0.5 |
| 3 | 191.057 | 1.996 | Quinic acid | C7 H12 O6 | 192.0643 | 2.68 | 92.75 | -4.68 |
| 4 | 133.0144 | 2.072 | Malic acid | C4 H6 O5 | 134.0217 | 0.05 | 90.84 | -0.97 |
| 5 | 164.0715 | 3.227 | L-Phenylalanine | C9 H11 N O2 | 165.0788 | 0.03 | 97.14 | 1.39 |
| 6 | 353.0882 | 3.767 | Chlorogenic Acid | C16 H18 O9 | 354.0955 | 0.29 | 94.38 | -1.05 |
| 7 | 339.0728 | 4.256 | Aesculin | C15 H16 O9 | 340.0804 | 0.46 | 92.17 | -2.8 |
| 8 | 353.0885 | 5.035 | Chlorogenic Acid | C16 H18 O9 | 354.0957 | 1.27 | 95.7 | -1.81 |
| 9 | 137.0246 | 5.462 | 2,5-Dihydroxybenzaldehyde | C7 H6 O3 | 138.0319 | 0.26 | 91.72 | -1.54 |
| 10 | 179.0358 | 6.441 | Caffeic Acid | C9 H8 O4 | 180.043 | 0.11 | 95.94 | -4.04 |
| 11 | 463.0884 | 7.935 | Quercetin 3-galactoside | C21 H20 O12 | 464.0956 | 1.03 | 97.99 | -0.34 |
| 12 | 431.099 | 11.086 | Apigenin 7-O-glucoside | C21 H20 O10 | 432.1062 | 0.04 | 96.97 | -1.37 |
| 13 | 445.0782 | 11.199 | Baicalin | C21 H18 O11 | 446.0856 | 1.35 | 93.75 | -1.56 |
| 14 | 193.051 | 12.668 | Scytalone | C10 H10 O4 | 194.0582 | 0.09 | 97.69 | -1.58 |
| 15 | 285.0404 | 15.003 | Luteolin | C15 H10 O6 | 286.0476 | 0.07 | 97.86 | 0.47 |
| 16 | 269.0458 | 17.489 | Apigenin | C15 H10 O5 | 270.0531 | 0.15 | 98.53 | -0.84 |
| 17 | 293.2127 | 30.622 | 9(S)-HOTrE | C18 H30 O3 | 294.22 | 1.31 | 97.93 | -1.72 |
| 18 | 275.2019 | 30.66 | Stearidonic Acid | C18 H28 O2 | 276.2092 | 0.22 | 94.58 | -0.92 |
| 19 | 291.1971 | 32.43 | 9-OxoOTrE | C18 H28 O3 | 292.2043 | 0.25 | 98.24 | -1.52 |
| 20 | 293.2125 | 34.062 | 9(S)-HOTrE | C18 H30 O3 | 294.2197 | 0.16 | 93.8 | -0.81 |
| 21 | 293.2123 | 34.928 | 9-OxoODE | C18 H30 O3 | 294.2195 | 0.14 | 98.39 | -0.01 |
| 22 | 293.2127 | 35.858 | 13(S)-HOTrE | C18 H30 O3 | 294.2199 | 0.34 | 90.94 | -1.35 |
| 23 | 297.2435 | 39.046 | cis-9,10-Epoxystearic acid | C18 H34 O3 | 298.2508 | 0.10 | 99.03 | -0.02 |
| 24 | 227.2016 | 40.766 | (+)-Isomyristic acid | C14 H28 O2 | 228.2089 | 0.09 | 97.11 | 0.33 |

**Supplementary Table S3 Compounds identified in the *Clerodendrum serratum (*Linn.) Moon (CSM) drying with dehydration chamber via positive electrospray ionization (ESI+) LC-MS analysis**

| No. | m/z | RT (min) | Name of the compound | Molecular Formulae | Molecular weight | %  Con  tent | Matching  Score | Diff (DB, ppm) |
| --- | --- | --- | --- | --- | --- | --- | --- | --- |
| 1 | 163.0389 | 9.205 | 4-Hydroxycoumarin | C9 H6 O3 | 162.03 | 2.20 | 90.87 | 0.2 |
| 2 | 449.1088 | 9.406 | Astragalin | C21 H20 O11 | 448.10 | 1.05 | 93.94 | -1.65 |
| 3 | 271.0597 | 11.063 | Apigenin | C15 H10 O5 | 270.05 | 0.72 | 93.44 | 1.34 |
| 4 | 447.0929 | 11.101 | Baicalin | C21 H18 O11 | 446.08 | 1.82 | 96 | -1.41 |
| 5 | 271.0601 | 17.478 | Genistein | C15 H10 O5 | 270.05 | 1.64 | 90.49 | 0.05 |
| 6 | 373.1987 | 25.701 | Lipoxin A5 | C20 H30 O5 | 350.20 | 0.40 | 92.9 | -0.3 |
| 7 | 293.2112 | 32.267 | 9-OxoOTrE | C18 H28 O3 | 292.20 | 0.41 | 91.44 | 0.17 |
| 8 | 496.3399 | 32.732 | 1-Palmitoyllysophosphatidylcholine | C24 H51 N O7 P | 496.34 | 0.55 | 92.73 | 0.08 |

**Supplementary Table S4 Compounds identified in the *Clerodendrum serratum* (Linn.) Moon (CSM) drying with dehydration chamber via positive electrospray ionization (ESI-) LC-MS analysis**

| No. | m/z | RT (min) | Name of the compound | Molecular Formulae | Molecular weight | %  Con  tent | Matching  Score | Diff (DB, ppm) |
| --- | --- | --- | --- | --- | --- | --- | --- | --- |
| 1 | 341.1102 | 1.79 | Sucrose | C12 H22 O11 | 342.1176 | 0.32 | 92.99 | -4.2 |
| 2 | 117.0193 | 2.669 | Succinic acid | C4 H6 O4 | 118.0266 | 0.07 | 93.23 | -0.03 |
| 6 | 445.0792 | 11.105 | Baicalin | C21 H18 O11 | 446.0865 | 0.42 | 90.56 | -3.6 |
| 7 | 609.1839 | 12.097 | Neohesperidin | C28 H34 O15 | 610.191 | 0.46 | 90.34 | -2.04 |
| 8 | 783.4551 | 17.056 | Astragaloside IV | C41 H68 O14 | 784.4621 | 1.06 | 91.24 | -1.57 |
| 9 | 191.072 | 19.466 | Ethyl-p-coumarate | C11 H12 O3 | 192.0793 | 0.09 | 97.19 | -3.3 |
| 11 | 269.083 | 28.204 | Cardamonin | C16 H14 O4 | 270.0902 | 0.20 | 93.69 | -3.51 |
| 12 | 291.198 | 32.234 | 9-OxoOTrE | C18 H28 O3 | 292.2053 | 2.48 | 94.97 | -4.94 |
| 13 | 227.2022 | 40.658 | (+)-Isomyristic acid | C14 H28 O2 | 228.2095 | 0.08 | 91.11 | -2.42 |

**Supplementary Table S5. MS/MS fragmentation characteristics used for putative identification**

| **Compound** | **Precursor m/z** | **Major fragment ions (m/z)** | **Relative intensity (%)** | **Annotation level** |
| --- | --- | --- | --- | --- |
| Astragalin (kaempferol-3-O-glucoside) | 449.108 | 287.055, 271.045 | 100, 45 | Putative |
| Kaempferol-7-O-glucoside | 449.108 | 287.055, 255.045 | 100, 38 | Putative |
