# Supplementary material for: Preparation and Evaluation of Niosomal Clerodendrum serratum (Linn.) Moon Extract Formulations: Comparative In Silico and In Vitro Studies of Drying Methods for the Treatment of Hemorrhoids
Source: Scientifica (Cairo). 2026 Apr 29;2026:3662572. doi: 10.1155/sci5/3662572 (PMC13126093; doi:10.1155/sci5/3662572)
Supplement: Supplementary file 1 — Supporting Information Additional supporting information can be found online in the Supporting Information section. [file SCI5-2026-3662572-s001.zip › SuppTableS3.pptx]

## Slide 1
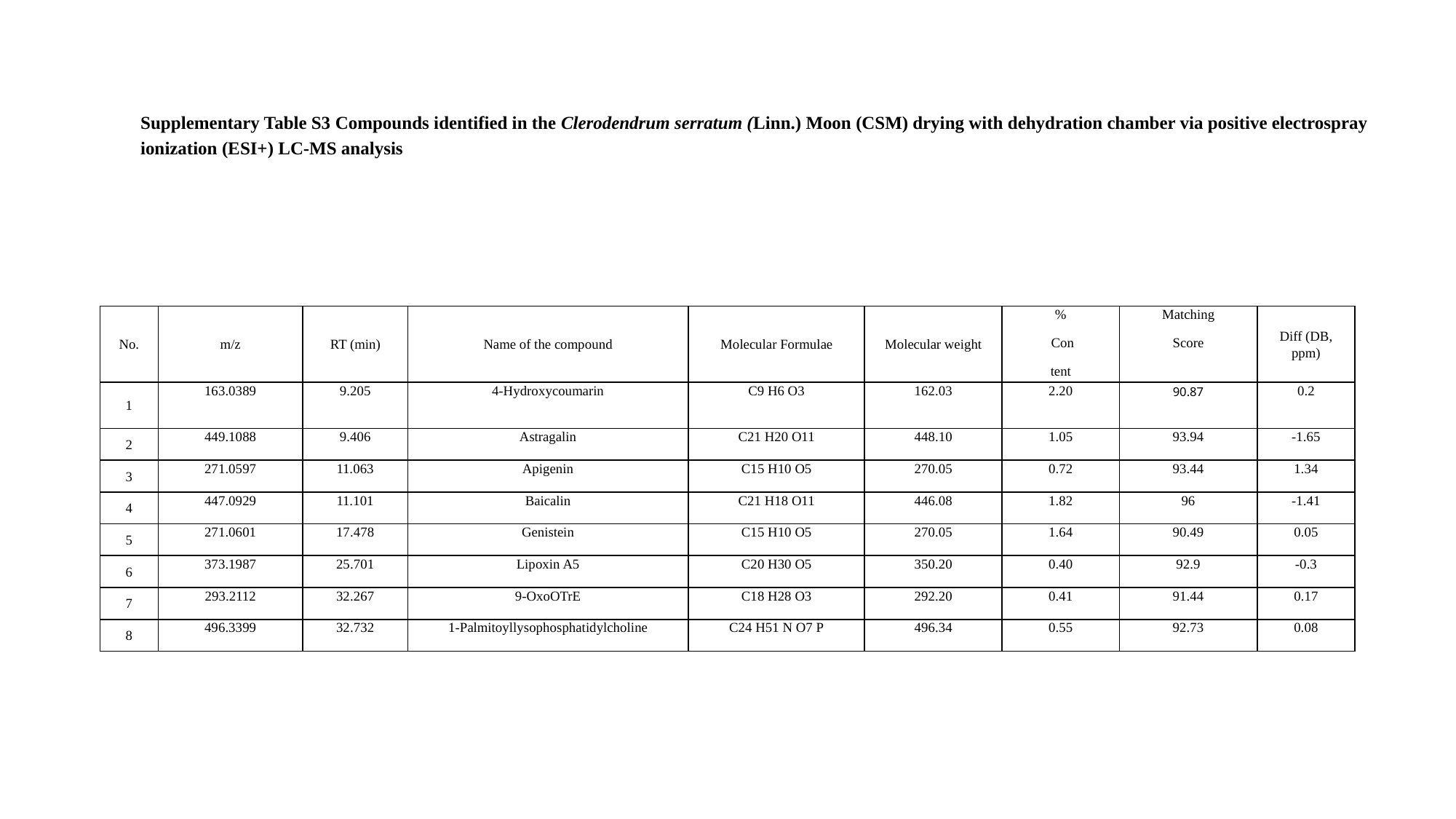

Supplementary Table S3 Compounds identified in the Clerodendrum serratum (Linn.) Moon (CSM) drying with dehydration chamber via positive electrospray ionization (ESI+) LC-MS analysis
| No. | m/z | RT (min) | Name of the compound | Molecular Formulae | Molecular weight | % Con tent | Matching Score | Diff (DB, ppm) |
| --- | --- | --- | --- | --- | --- | --- | --- | --- |
| 1 | 163.0389 | 9.205 | 4-Hydroxycoumarin | C9 H6 O3 | 162.03 | 2.20 | 90.87 | 0.2 |
| 2 | 449.1088 | 9.406 | Astragalin | C21 H20 O11 | 448.10 | 1.05 | 93.94 | -1.65 |
| 3 | 271.0597 | 11.063 | Apigenin | C15 H10 O5 | 270.05 | 0.72 | 93.44 | 1.34 |
| 4 | 447.0929 | 11.101 | Baicalin | C21 H18 O11 | 446.08 | 1.82 | 96 | -1.41 |
| 5 | 271.0601 | 17.478 | Genistein | C15 H10 O5 | 270.05 | 1.64 | 90.49 | 0.05 |
| 6 | 373.1987 | 25.701 | Lipoxin A5 | C20 H30 O5 | 350.20 | 0.40 | 92.9 | -0.3 |
| 7 | 293.2112 | 32.267 | 9-OxoOTrE | C18 H28 O3 | 292.20 | 0.41 | 91.44 | 0.17 |
| 8 | 496.3399 | 32.732 | 1-Palmitoyllysophosphatidylcholine | C24 H51 N O7 P | 496.34 | 0.55 | 92.73 | 0.08 |
