# Supplementary figures and images for: Preparation and Evaluation of Niosomal Clerodendrum serratum (Linn.) Moon Extract Formulations: Comparative In Silico and In Vitro Studies of Drying Methods for the Treatment of Hemorrhoids
Source: Scientifica (Cairo). 2026 Apr 29;2026:3662572. doi: 10.1155/sci5/3662572 (PMC13126093; doi:10.1155/sci5/3662572)

## Slide 1
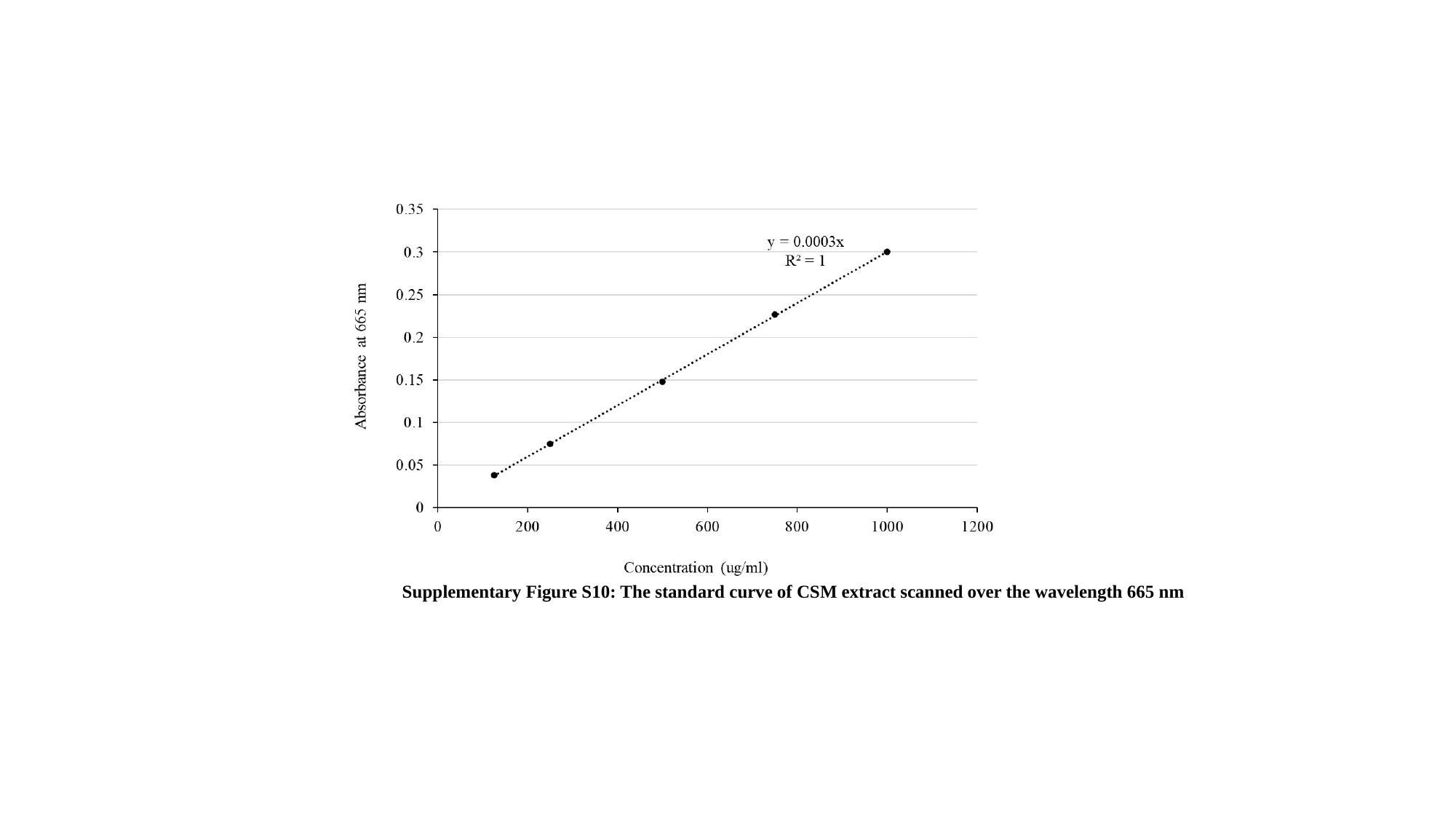

Supplementary Figure S10: The standard curve of CSM extract scanned over the wavelength 665 nm

Supplement: Supplementary file 1 — Supporting Information Additional supporting information can be found online in the Supporting Information section. [file SCI5-2026-3662572-s001.zip › SuppFig10.pptx]

## Slide 1
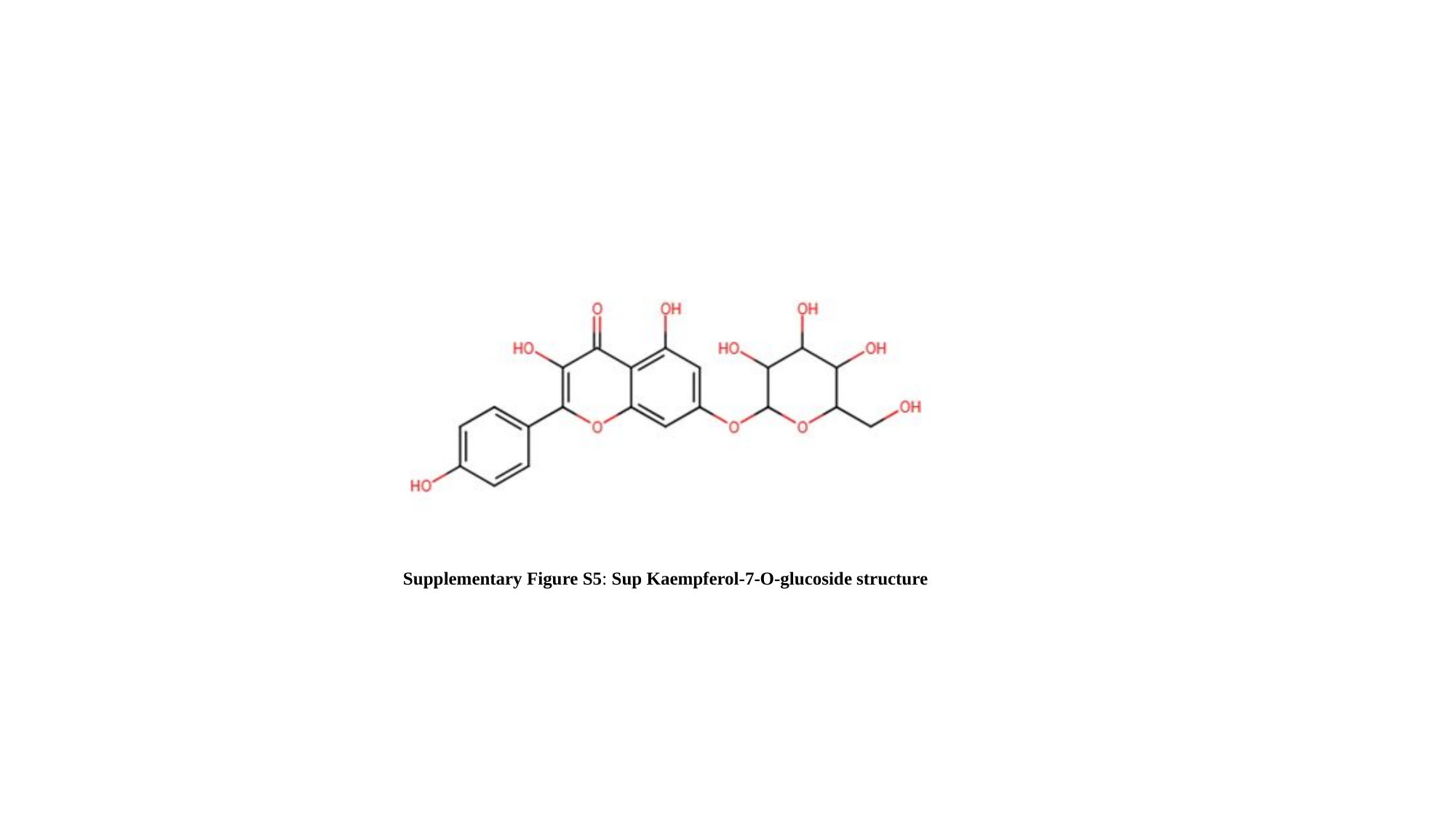

Supplementary Figure S5: Sup Kaempferol-7-O-glucoside structure

Supplement: Supplementary file 1 — Supporting Information Additional supporting information can be found online in the Supporting Information section. [file SCI5-2026-3662572-s001.zip › SuppFig5.pptx]

## Slide 1
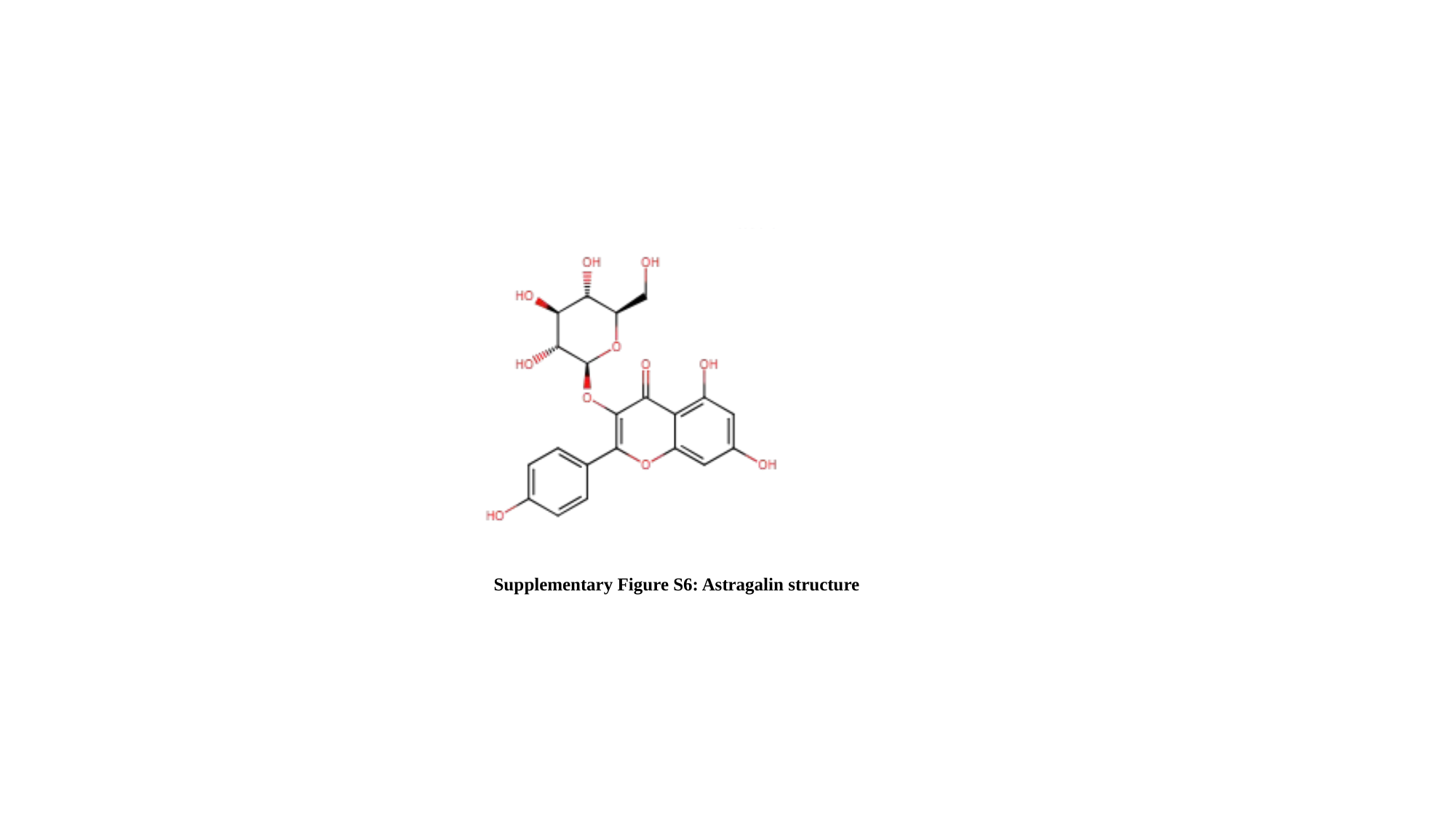

Supplementary Figure S6: Astragalin structure

Supplement: Supplementary file 1 — Supporting Information Additional supporting information can be found online in the Supporting Information section. [file SCI5-2026-3662572-s001.zip › SuppFig6.pptx]

## Slide 1
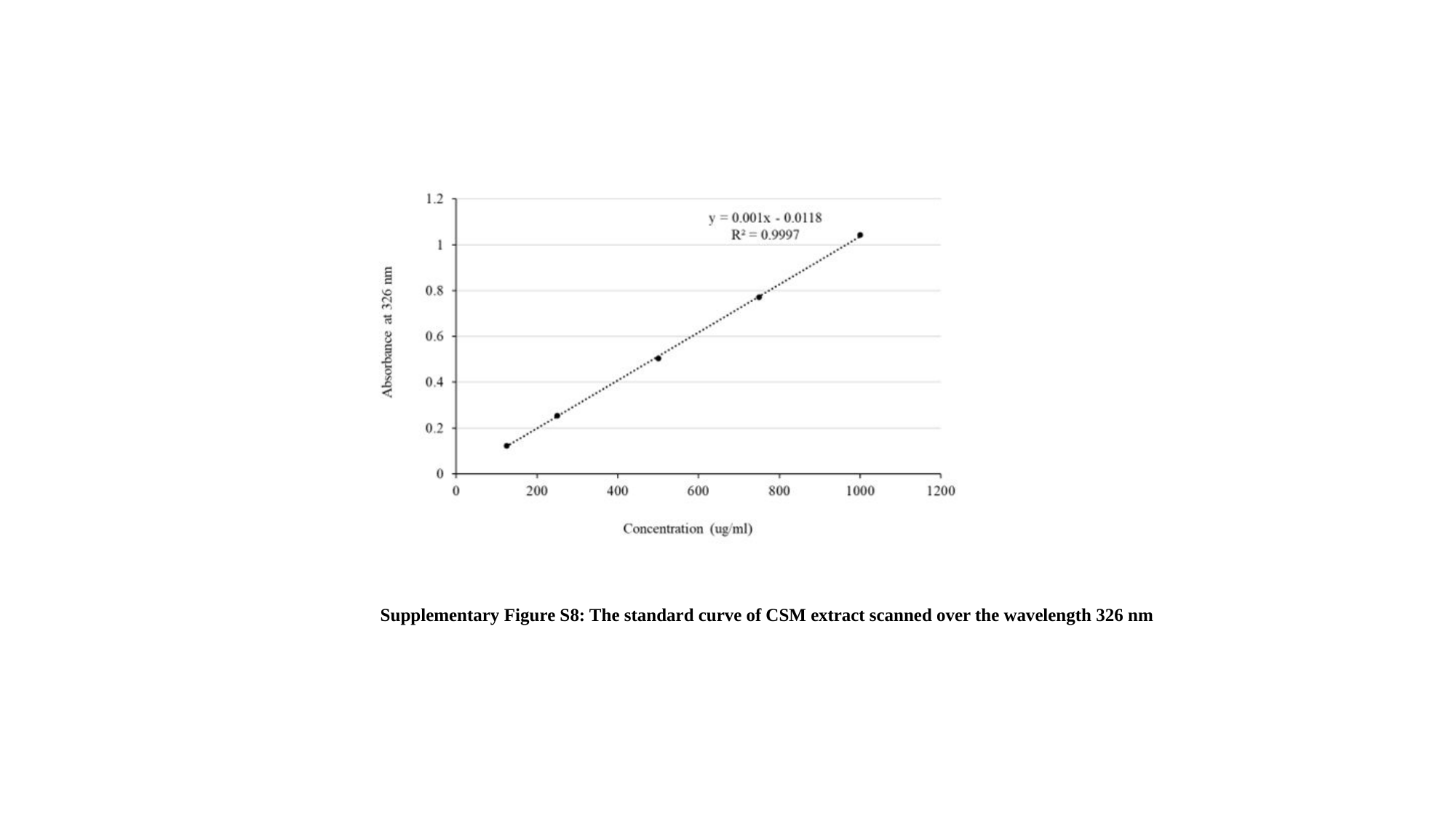

Supplementary Figure S8: The standard curve of CSM extract scanned over the wavelength 326 nm

Supplement: Supplementary file 1 — Supporting Information Additional supporting information can be found online in the Supporting Information section. [file SCI5-2026-3662572-s001.zip › SuppFig8.pptx]

## Slide 1
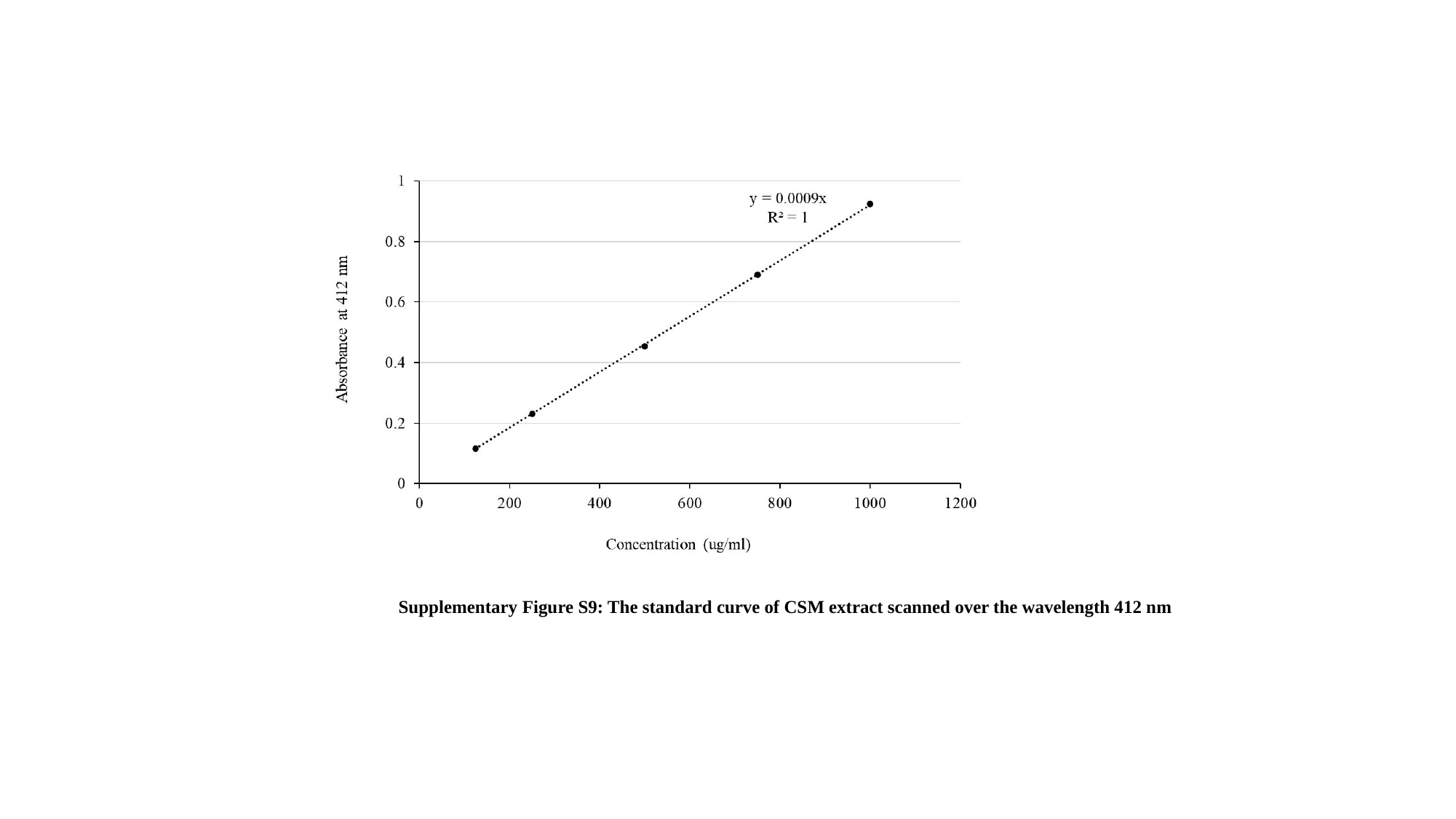

Supplementary Figure S9: The standard curve of CSM extract scanned over the wavelength 412 nm

Supplement: Supplementary file 1 — Supporting Information Additional supporting information can be found online in the Supporting Information section. [file SCI5-2026-3662572-s001.zip › SuppFig9.pptx]
